# Co-creating a patient-driven digital monitoring agenda for thoracic aortic disease: a multiphase qualitative study

**DOI:** 10.1136/bmjopen-2026-121086

**Published:** 2026-09-25

**Authors:** Nimrat Grewal, Nora Bacour, Youness Aouragh, Aytug U Tirpan, Anne M Beukers, Simran Grewal

**Affiliations:** 1 Department of Cardiothoracic Surgery, Amsterdam UMC Locatie AMC, Amsterdam, Netherlands; 2 Department of Anesthesiology, Amsterdam UMC Locatie AMC, Amsterdam, North Holland, Netherlands; 3 Department of Orthopaedic Surgery, Onze Lieve Vrouwe Gasthuis, Amsterdam, Netherlands

**Keywords:** QUALITATIVE RESEARCH, SURGERY, Patient Participation

## Abstract

**Abstract:**

**Objectives:**

To identify, through structured co-creation with patients across the thoracic aortic disease (TAD) spectrum, which domains of between-visit monitoring patients themselves consider meaningful and to translate these into a patient-driven digital monitoring agenda.

**Design:**

Qualitative co-creation study in four sequential phases: secondary analysis of existing patient engagement material (A), targeted focus groups addressing under-represented perspectives (B), a mixed-stakeholder co-design workshop (C) and a member-check validation session (D). Analysis used reflexive thematic analysis and framework synthesis. Reported following Consolidated Criteria for Reporting Qualitative Research (COREQ).

**Setting:**

A single Dutch tertiary referral centre for TAD, 2024–2025.

**Participants:**

Adults (≥18 years) with TAD, purposively sampled across three trajectories: surgically managed acute dissection, elective aortic surgery and aneurysm or bicuspid aortic valve under surveillance. Phase A, 45 participants in six sessions; phase B, 13 in two focus groups; phase C, 9 (6 patients, 3 healthcare professionals); phase D, 9 participants from earlier phases.

**Outcome measures:**

Patient-derived domains considered relevant to digital monitoring and their translation into components of a monitoring agenda, together with the conditions participants regarded as necessary for such monitoring to be acceptable.

**Results:**

Five domains recurred across trajectories: managing uncertainty and fear of recurrence, interpreting symptoms and bodily signals, blood pressure self-monitoring and variability, life participation including activity and reproductive decisions and accessibility and proportionality. These were translated into an agenda comprising symptom contextualisation, longitudinal blood pressure trends, patient-reported outcomes, contextual activity feedback, educational decision support and transparent escalation pathways. Participants framed monitoring as an adjunct to imaging surveillance, not a replacement, emphasising interpretability and restrained alerting.

**Conclusion:**

Structured patient engagement and iterative co-design produced a patient-driven digital monitoring agenda for TAD integrating biomedical and psychosocial dimensions, describing priorities articulated by these participants rather than their distribution and providing a foundation for developing and evaluating hybrid digital surveillance models in aortic care.

**STRENGTHS AND LIMITATIONS OF THIS STUDY**

- The four-phase design combined secondary analysis of existing engagement material, targeted focus groups, a mixed-stakeholder co-design workshop and a member-check validation session, so that findings were developed and tested iteratively rather than generated in a single round of data collection.
- Purposive sampling across three clinical trajectories (acute dissection, elective surgery and imaging surveillance) allowed comparison of experiences that are usually studied separately, and phase B was added specifically to recruit perspectives that were under-represented in phase A.
- Analysis used reflexive thematic analysis, with two researchers coding separately before comparing readings in team discussion, documented reflexivity concerning the facilitators’ dual clinical and research roles and member-check validation of the resulting framework.
- Sessions were not audio-recorded, and no verbatim transcripts were produced, so the findings rest on material written by participants during the sessions, on statements recorded contemporaneously on flip charts and on field notes and are reported as summary rather than as quoted speech.
- All participants were recruited from a single tertiary referral centre, and no clinician-only group was convened, so the findings may over-represent patients who are actively engaged in specialist follow-up and do not permit a formal comparison of patient-derived with clinician-derived priorities.

## Introduction

 Thoracic aortic disease (TAD) encompasses a spectrum of conditions, including thoracic aortic aneurysm, genetically mediated aortopathies, bicuspid aortic valve associated dilation and acute aortic dissection.^1–3^ Advances in surgical techniques and perioperative management have improved survival following acute TAD events.^4 5^ Consequently, a growing population of individuals now lives with chronic aortic disease, requiring lifelong imaging surveillance, medical therapy and risk stratification.^6 7^

Despite structured imaging follow-up, many patients report persistent uncertainty regarding symptom interpretation, blood pressure management and safe participation in daily activities.^8 9^ In particular, individuals who have experienced acute aortic dissection often describe sustained hypervigilance and fear of recurrence.^10^ Conversely, patients under surveillance without prior events may experience prolonged anticipatory anxiety during years of imaging follow-up.

Digital health technologies, including home blood pressure monitoring, wearable activity devices and patient portals, offer opportunities to complement traditional surveillance.^11 12^ However, current digital initiatives in cardiovascular care are largely clinician-driven and focused on physiological parameters. Little is known about how individuals with TAD conceptualise digital monitoring, which domains they perceive as meaningful, and how such tools might affect anxiety, autonomy and health equity.

We therefore aimed to develop a patient-driven digital monitoring agenda for TAD using a structured, multiphase qualitative co-creation approach embedded within routine clinical care. This process involved patients across acute, elective and surveillance pathways, as well as multidisciplinary clinicians engaged in aortic care. An overview of the study design, the resulting patient-derived domains and the monitoring agenda is presented in figure 1.

**Figure 1:**
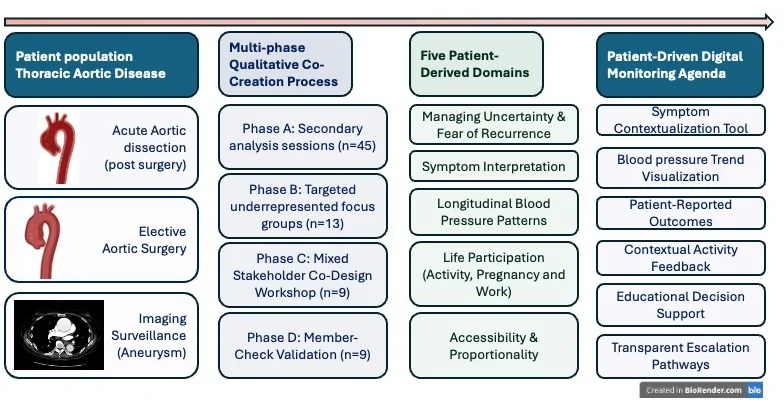
Overview of the multiphase co-creation process, the resulting patient-derived domains and the patient-driven digital monitoring agenda for thoracic aortic disease. The left-hand column shows the three clinical trajectories from which participants were purposively sampled (surgically managed acute aortic dissection, elective aortic surgery and thoracic aneurysm under imaging surveillance). The second column shows the four sequential study phases with the number of participants in each. The third column shows the five patient-derived domains identified through reflexive thematic analysis. The right-hand column shows the six components of the resulting digital monitoring agenda. Created with BioRender.com.

## Methods

### Ethics approval and consent to participate

All participants received written and verbal information regarding the study aims, procedures and use of anonymised data. Written informed consent was obtained prior to participation in newly conducted focus groups and workshops. For previously conducted patient engagement sessions included in phase A, only materials for which participants had consented to pseudonymised research use were analysed.

Data were pseudonymised prior to analysis. Identifying information was removed from transcripts and field notes. All data were stored on secure institutional servers accessible only to the research team.

### Patient and public involvement

Patients were actively involved throughout the study. Earlier co-creation sessions informed the research focus (phase A). Additional focus groups were conducted to address under-represented perspectives (phase B). Patients participated in a co-design workshop translating findings into a digital monitoring framework (phase C), and findings were discussed during a member-check validation session (phase D). Patients therefore contributed to the design, refinement and validation of the proposed digital monitoring agenda.

### Study design and setting

We performed a multiphase qualitative study within a Dutch tertiary aortic referral centre, in 2024–2025. The study was designed as an iterative co-creation process comprising four phases. In the first phase (phase A) a secondary analysis was performed of previously conducted patient engagement and co-creation sessions within clinical programmes. These materials were explored to identify experiences and needs related to digital monitoring. The second phase (phase B) targeted focus groups to address under-represented perspectives. In the third phase (phase C) a mixed stakeholder co-design workshop was held, translating thematic findings into a practical monitoring framework. The last phase (phase D) included a member-check validation session. The overall design is summarised in figure 1.

The study adhered to the Consolidated Criteria for Reporting Qualitative Research (COREQ)^13^; the completed checklist is provided as online supplemental file 1.

### Research team and reflexivity

Focus groups and workshops were facilitated by NG (MD, PhD), cardiothoracic surgeon and SG (MD, PhD), orthopaedic surgeon. Both facilitators were involved in clinical practice and translational research at the time of the study. The facilitators were female and had prior experience conducting qualitative interviews and co-creation sessions within clinical care programmes.

Participants were familiar with the clinical setting, but no prior research relationship was established before study participation. Participants were informed that the aim of the study was to explore patient perspectives to inform future digital monitoring strategies.

The facilitators were aware that their clinical roles could shape what participants felt able to say, and reflexivity was addressed prospectively rather than only during analysis. Three issues were identified and documented in a reflexive log kept throughout the study. First, because NG is a cardiothoracic surgeon at the centre where participants receive their care, there was a risk that participants would withhold criticism of the follow-up they had received; each session therefore opened with an explicit statement that the purpose was to inform future care, that nothing said would affect the participant’s own treatment, and that critical views were particularly welcome, and NG did not facilitate discussion with participants for whom she was the operating surgeon. Second, both facilitators are clinicians with a prior interest in remote monitoring, which risked steering discussion toward the feasibility of technology rather than toward whether monitoring was wanted at all; this became visible when early coding produced a disproportionate number of feature-oriented codes, and the topic guide was subsequently reordered so that questions about experience and unmet need preceded any mention of digital tools. Third, the initial coding was performed by the research team who had not facilitated the sessions, and their readings were compared with those of the facilitators specifically to surface interpretations that reflected clinical framing rather than participants’ accounts; the decision to retain ‘proportionality and trust’ as a domain in its own right, rather than treating it as a design requirement subordinate to the clinical domains, followed from one such discussion.

### Participants and sampling

Adult patients (≥18 years) diagnosed with TAD were eligible. We purposively sampled individuals across three clinical trajectories:

1. Surgically managed acute aortic dissection.
2. Elective aortic surgery for aneurysm with or without valve intervention.
3. Thoracic aneurysm or bicuspid aortic valve under imaging surveillance.

We aimed to ensure diversity in age, sex, treatment pathway and time since diagnosis. For phase B, targeted recruitment was undertaken to enrich perspectives that were less represented in earlier sessions:

- Women across reproductive and post-reproductive life stages.
- Participants with varying digital literacy and socioeconomic backgrounds.

Recruitment occurred during outpatient visits and via follow-up contact. Participation was voluntary. No participants withdrew after providing consent. Recruitment continued until thematic sufficiency was reached across groups.

Rather than fixing a sample size in advance, we specified stopping criteria before data collection in phase B and framed these in terms of thematic sufficiency, understood as information power, rather than as data saturation in the inductive-inferential sense.

Three criteria were defined a priori: (1) that all three clinical trajectories were represented and that the perspectives identified as under-represented in phase A had been purposively recruited; (2) that two consecutive sessions yielded no new codes that changed the content or the boundaries of the candidate domains; and (3) that the research team judged the material sufficient to allow each domain to be translated into concrete monitoring components in phase C. These criteria were reviewed by the research team after every session. All three were met after the second focus group in phase B, and recruitment was closed at that point. We use the term thematic sufficiency in preference to saturation because the analytical aim was to develop an agenda that was adequately informed across trajectories, not to demonstrate that no further themes could ever be identified.

Participant characteristics are summarised by study phase in table 1. In line with the journal’s policy on participant privacy, characteristics are reported in aggregate only; no individual-level rows are presented, and age is reported as a range.

**Table 1:** Characteristics of participants by study phase

Characteristic	Phase A (n=45)	Phase B (n=13)	Phase C (n=9)	Phase D (n=9)
Age, years — median (range)	56 (45–65)	54 (45–65)	57 (46–64)	56 (47–65)
Women — n	20	9	4	5
Clinical trajectory — n				
Acute aortic dissection, surgically managed	15	4	2	3
Elective aortic surgery (±valve intervention)	15	4	2	3
Aneurysm/bicuspid aortic valve under surveillance	15	5	2	3
Time since diagnosis or index event — n				
<2 years	18	5	2	3
2–5 years	18	5	3	4
>5 years	9	3	1	2
Healthcare professionals — n	0	0	3	0
Sessions	Six engagement sessions	Two focus groups	One co-design workshop	One online validation session

Data are numbers of participants unless stated otherwise. Characteristics are reported in aggregate by phase, and age as median (range), in accordance with the journal’s policy on identifiability in small populations. Clinical trajectory and time-since-event rows describe patient participants only, so the phase C column sums to six rather than nine. Participants are not independent between phases: all nine phase D participants had taken part earlier, and columns should not be summed. Age and sex are likewise reported for patient participants only; because participants overlapped between phases, the phase columns should not be summed to a total number of individuals.

### Data collection

#### Phase A: analysis of existing engagement sessions

Multiple patient engagement and co-creation sessions were conducted between 2024 and 2025, as part of care pathway development and educational initiatives. These co-creation sessions included individuals following acute dissection, elective surgery and surveillance programmes. Available materials (field notes, anonymised transcripts, facilitator summaries) were collated for analysis. Phase A included six engagement sessions involving 45 unique participants across the spectrum of TAD.

The nature of the record shaped what can be reported, and we state it rather than leave it to be inferred. The sessions were not audio-recorded, and no verbatim transcripts were produced. The material falls into three kinds. First, text written by participants themselves: single words and short phrases on cards and post-it notes, and worksheets on which participants set out what had changed for them between the period before their event and the present. Second, statements written on flip charts by the facilitator during discussion, as they were spoken; the chart was visible to the group as it was filled in, so participants could see, correct and add to what was recorded at the time. Third, field notes and facilitator summaries. Table 2 sets out the sessions in each phase, the prompt used in each and the form the material took.

**Table 2:** Sessions by study phase: participant group, prompt and form of the record

Phase/ session	Participant group	n	Prompt used	Form of the record
A1	Acute aortic dissection, post-surgery	8	What living with your aorta since the operation means to you	Individual cards; group flip chart
A2	Acute aortic dissection, recovery	7	What you did, thought or felt differently before the dissection than now	Individual before/now worksheets
A3	Elective aortic surgery	8	What you ran into at home that you had not anticipated before the operation	Individual cards, then group flip chart
A4	Thoracic aneurysm/bicuspid aortic valve under imaging surveillance	7	What happens for you between two scans (timeline: after scan → months in between → next scan approaching → result)	Group flip-chart timeline
A5	Mixed (acute, elective, surveillance)	8	When measuring your blood pressure helps you and when it does not	Two group flip charts
A6	Mixed (acute, elective, surveillance)	7	What support between hospital appointments should and should not do	Individual post-it notes, then clustering by participants
B1	Women across life stages	7	Moments at which the condition affects the choices you make; where you do and do not get answers	Individual cards; group flip chart
B2	Accessibility and digital literacy	6	What makes digital care easy or difficult for you; how to make it usable for everyone	Individual cards; group flip chart
C	Mixed stakeholder co-design workshop (six patients, three healthcare professionals)	9	Translating findings from phases A and B into components; prioritisation by sticker vote; mapping what should happen when something deviates	Post-it notes on finding cards; ranked flip chart; jointly drawn escalation pathway
D	Member-check validation	9	For each domain: recognised/uncertain/missing; when the proposed system would help and when it would work against you	Individual cards; group flip chart

Prompts are given in English translation and represent the opening task of each session rather than the only question asked; the full guide is provided as online supplemental file 2. Cards, post-it notes and worksheets denote text written by participants themselves; group flip chart denotes items collected from the group by the facilitator during the session. Flip-chart material in the form of word lists is reproduced in online supplemental file 3; continuous sentences recorded from discussion are not reproduced. No session was audio-recorded and no verbatim transcripts were produced.

#### Phase B: targeted focus groups

Two additional focus group sessions were organised. The first group comprised women reflecting on disease impact across life events, including pregnancy, caregiving and work participation. The second group comprised participants with varying digital literacy and socioeconomic backgrounds. The women’s focus group included seven participants, while the focus group addressing accessibility and digital literacy included six participants.

Each session lasted approximately 90–120 min and followed a semi-structured guide exploring experiences with follow-up, perceptions of digital monitoring and concerns regarding alerting, data sharing and autonomy. Focus groups were conducted in dedicated meeting rooms at the Amsterdam University Medical Center. The member-check validation session was conducted online via a secure institutional platform. No non-participants were present during focus groups or workshops.

The sessions followed a semi-structured guide of 35 questions covering eight areas: living with the condition; uncertainty and symptoms; the period around check-ups and scans; blood pressure; return to daily life; support between hospital visits; digital support; and a closing invitation to raise anything not asked about. Questions about experience and unmet need were placed before any question about digital tools, so that participants set out what they needed before the possibility of technology was introduced. The guide was used flexibly: not every question was put in every session, the order followed the discussion and facilitators followed-up on what participants raised rather than working through the list. The full guide is provided, in Dutch with an English translation, as online supplemental file 2.

The guide was drafted jointly by NG and SG and was revised by the full research team before use. Three sources fed into it: the questions patients most often raise in aortic follow-up consultations at our centre; the themes identified in our earlier work on life impact, activity, sleep and quality of life after acute aortic dissection^8 14^; and the uncertainty, symptom appraisal and psychological burden described in the wider aortopathy literature.^10 15^ Questions were written in lay Dutch, grouped into the eight areas listed above, and ordered so that experience and unmet need were covered before digital tools were introduced. The guide was not pilot tested in a separate session outside the study; the first phase B focus group served as its first use, no changes to wording or order were required afterwards and the same guide was used in both phase B focus groups. Its development is also described in online supplemental file 2.

#### Phase C: co-design workshop

A structured workshop brought together nine participants: six patients, purposively selected to represent all three clinical trajectories, and three healthcare professionals representing cardiology, cardiothoracic surgery and clinical genetics, cardiothoracic surgery being represented by a nurse specialist. Using facilitated small-group exercises, participants worked from cards summarising the principal findings of phases A and B to identify what might help between hospital appointments, ranked the resulting ideas by vote and jointly mapped the sequence that should follow when a measurement or symptom deviates.

#### Phase D: member-check validation

A summary framework was presented in an online validation session. Participants were invited to comment on clarity, completeness and potential unintended consequences. The validation session included nine participants who had taken part in earlier phases of the study.

Individual transcripts were not returned for correction, because none were produced. Participant checking therefore took two other forms: within the sessions, the flip-chart record was written in front of the group and remained visible, so participants could correct or extend it as it was written and in session 6 the post-it notes were clustered by participants themselves; at the level of the synthesised findings, the framework was returned to participants in phase D. Checking a synthesis is a weaker safeguard than checking a record: it can confirm that a framework is recognisable, but cannot recover a statement compressed or lost when first written down.

### Data analysis

Data were analysed using reflexive thematic analysis as described by Braun and Clarke.^16 17^ Two researchers each familiarised themselves with the data through repeated reading of the available material. Initial codes were generated inductively and refined iteratively through discussion within the research team. All developed codes were then grouped into candidate themes, which were reviewed and adapted in relation to the full dataset. Data were managed and analysed without dedicated qualitative data analysis software.

Phase A material was generated before this study was designed, under prompts that differed from those used later and some phase A participants also took part in later phases. It was therefore used to identify candidate domains and to establish which perspectives were under-represented, which is what phase B was then recruited to address, rather than as an independent source of corroboration. Every finding reported in the Results is attributed to the phase and session it rests on, and table 2 states the prompt used in each session, so that the material behind each claim can be identified. Because participants overlapped between phases, recurrence of a domain across phases is reported as recurrence within one connected group rather than as replication in independent samples.

After identifying the main themes, we explored how these experiences could inform digital monitoring in practice, meaning revisiting the themes and exploring what participants suggested about everyday needs, uncertainties and points where additional support might be helpful. Through this process, themes were gradually linked to possible elements of a monitoring approach.

No codebook or predetermined coding framework was used, and no coding tree was retained. This follows from the analytical approach: in reflexive thematic analysis coding is an interpretive act rather than a procedure standardised across coders, and demonstrating coding reliability is not held to be a meaningful quality criterion.^16 17^ The two coders therefore coded separately and then compared readings in team discussion in order to widen the interpretive range rather than to establish agreement; no agreement statistic was calculated. Codes were grouped into candidate themes through successive discussions, and the five domains were arrived at by collapsing and re-testing those candidates against the full dataset. Because no fixed framework was retained we cannot supply a coding tree; the relationship between domains and agenda components is set out instead in table 3.

**Table 3:** Mapping of patient-derived domains onto components of the digital monitoring agenda

Patient-derived domain	Core patient concern	Component of the digital monitoring agenda	Design conditions specified by participants
Managing uncertainty and fear of recurrence	Uncertainty is continuous after dissection and cyclical around imaging in surveillance; reassurance is time-limited	Patient-reported outcomes covering well-being and anxiety, timed to the surveillance cycle	Must reduce ambiguity rather than generate further data; optional rather than obligatory completion
Interpreting symptoms and bodily signals	Difficulty judging whether a sensation is clinically relevant, and difficulty describing it to a clinician	Symptom contextualisation tool structuring duration, progression, associated features and comparison with previous episodes	Structured reflection, not automated triage; output intended to support the consultation, not replace it
Longitudinal blood pressure patterns	Single readings drive repeated measurement and anxiety; participants reason in trends, not thresholds	Longitudinal blood pressure trend visualisation with contextual framing	Trend display prioritised over isolated values; consistent guidance on measurement frequency and on what constitutes meaningful change
Life participation (activity, pregnancy, work)	Generic advice does not answer ‘what is safe for me’; reproductive and occupational guidance arrives fragmented across specialties	Contextual activity feedback and educational decision-support modules tailored to life stage	Optional and confidence-building rather than restrictive; coherent across specialties
Accessibility and proportionality	Frequent or vague alerts increase tension; access barriers exclude; storage without use erodes trust	Transparent escalation pathways embedded in existing care pathways	Every alert linked to a named action, contact and time frame; plain language and low technical threshold; explicit statement of who reviews data and how often

Domains were derived inductively from the material generated in phases A and B; components were developed from them in the phase C co-design workshop and confirmed in phase D. The fourth column gives the conditions participants attached to each component. The mapping is one of emphasis rather than exclusivity: several components address more than one domain.

These interpretations were discussed within the research team and revisited during member-check sessions. This helped to keep the emerging framework grounded in participants’ accounts and to ensure that the translation from experience to practical components remained faithful to the data.

Two conventions follow for how the findings are reported. First, nothing in the Results appears in quotation marks. Cards, post-it notes and flip-chart collections were produced in response to prompts of the form ‘write two or three words describing what living with your aorta since the operation means to you’; what a participant writes under such a prompt is a contribution to a structured exercise rather than a statement offered about their life and setting it in quotation marks would attribute a communicative intent that was not present. The statements written on flip charts as continuous sentences during discussion sit closer to speech, but they are the facilitator’s compression of what was said, written at speed and presenting them as quotations would imply a fidelity the method cannot support; they are therefore not reproduced. The Results report what the material shows, in our own words. Second, and so that the basis of each finding can be inspected rather than taken on trust, the complete output of every elicitation exercise is reproduced under the prompt that generated it in online supplemental file 3, in the original Dutch with an English translation. These lists are anonymous by construction: items were pooled during the sessions without recording which participant contributed which item, and no key exists that would permit attribution. Complete lists are given rather than selections, so that the reader sees the full distribution of what each exercise produced. Each finding in the Results is attributed to the phase and session that generated it, and table 2 states the prompt used in each session and the form of the resulting record. Translation was carried out by a bilingual member of the team and checked against the original by a second.

## Results

Across clinical trajectories, participants did not describe digital monitoring as a replacement for imaging surveillance. Instead, it was framed as something that might fill the space between appointments, the long intervals during which questions accumulate and reassurance gradually fades.

Although experiences differed depending on whether participants had undergone acute dissection, elective surgery or were in surveillance without prior events, several recurrent patterns emerged. For each domain we indicate below which phase and session the finding rests on; the complete output of every exercise, under the prompt that generated it, is reproduced in online supplemental file 3.

### Living with ongoing uncertainty

Uncertainty was not episodic; it was described as structural to living with TAD. Participants following acute dissection spoke about a heightened awareness of their body. Sensations that would previously have been ignored, transient chest discomfort, muscular pain, fatigue, now required interpretation. Several described an internal negotiation: whether to dismiss a symptom as benign or escalate it. In the same material, and often from the same participants, gratitude and a sense of having been given a second chance appeared alongside this watchfulness rather than in place of it (phase A, sessions 1 and 2).

In contrast, participants under imaging surveillance described uncertainty as cyclical. Anxiety often increased in the weeks preceding imaging, followed by temporary relief when results were stable. However, reassurance was time limited. The absence of symptoms did not necessarily equate to a sense of safety. Mapped onto a timeline, the weeks before a scan were characterised by disturbed sleep, by renewed attention to the aortic diameter and to previous results and by independent searching for information; participants noted that a scan reassures but that the reassurance wears off as the next one approaches (phase A, session 4).

Digital monitoring was seen as potentially helpful if it reduced ambiguity. However, participants were cautious. Monitoring that simply produced more numbers, without interpretation, was perceived as likely to intensify vigilance rather than relieve it.

What emerged was not a request for constant monitoring, but for calibrated support.

### Symptom interpretation as a shared task

A recurring difficulty concerned symptom appraisal. Participants struggled to determine when pain or discomfort was clinically relevant. This uncertainty sometimes led to delayed consultation; at other times, to repeated urgent evaluations later deemed unnecessary.

Importantly, participants did not ask for automated triage. Instead, they described a need for structured reflection: guidance that might help them articulate duration, progression, associated features and comparison with prior experiences.

Several noted that the process of describing symptoms coherently to a clinician was itself challenging. A digital tool was envisioned as a way to organise thoughts before contact, rather than as a substitute for professional evaluation.

### Blood pressure measurements

Home blood pressure monitoring was common across participants, yet it carried mixed meanings.

For some of the participants, measuring blood pressure at home brought a sense of involvement and control. It was something tangible they could do, a way to feel engaged in their own follow-up. Several described it as reassuring to see the values themselves and confirm that their condition was stable.

For other participants, particularly those who had experienced an acute dissection, blood pressure measurement was more complicated. Readings taken during moments of stress sometimes triggered a cascade: one elevated value led to repeated checks, followed by rising anxiety rather than reassurance. A single higher measurement could dominate attention, even when subsequent readings were normal and the tension generated by repeated measurement became itself a reason the next reading was raised. Some participants described deliberately not measuring on days when they judged it would unsettle them (phase A, sessions 2 and 5).

Participants seemed to value the pattern most, rather than the individual numbers. They spoke about wanting to understand how their blood pressure behaves over time: whether it is stable, drifting or fluctuating in a way that matters. A visual overview of trends was described as more helpful than reacting to isolated values.

There was also uncertainty about how often to measure and when to act. Some participants reported receiving different advice from different clinicians. Clearer and more consistent guidance, ideally integrated within a digital system, was viewed as important to prevent both undermeasurement and overmeasurement.

Interestingly, several participants referred spontaneously to variability, to ups and downs across days or weeks, suggesting that they already think in terms of patterns rather than single thresholds. Any future digital monitoring approach will need to acknowledge this lived understanding of fluctuation over time.

### Returning to life

Monitoring was almost never discussed as a standalone concept. It was always tied to everyday life.

Participants spoke at length about physical activity. Not in abstract terms, but very concrete: walking uphill, cycling against the wind, lifting groceries, returning to the gym. The question was rarely what guidelines permit in general, but how a person is to know what is safe for them in particular. General recommendations were familiar, yet many described missing personal context, how advice applied to their own body, their own history.

Women described additional layers of uncertainty around pregnancy and family planning. Information was often received in fragments, a comment during a surgical consultation, another during cardiology follow-up, sometimes differing in tone. Several expressed a need for coherent guidance that acknowledges both medical risk and personal life plans. A digital tool was imagined as a place where such information could be brought together, rather than scattered across appointments.

In the focus group convened with women across life stages (phase B), reproductive and life-course concerns were the dominant content. Participants distinguished sharply between what their follow-up reliably answers, aortic diameter, scan results, blood pressure, medication, the timing of the next appointment, the indication for surgery and what remains unresolved. The unresolved list was longer and more consequential: whether pregnancy is safe, who should raise it and when, whether they may lift their own children, which medications are compatible with a wish to conceive, what the condition means at menopause, how to combine work with recovery, whether the condition is heritable, what to tell their children and whether general advice applies to them at all. Participants described receiving each part of this from a different clinician and none of it as a whole, and asked for guidance matched to their stage of life rather than to their aortic dimensions alone.

Work and caregiving responsibilities were equally prominent. Participants wanted to resume ordinary roles without being constantly reminded of vulnerability. Some described the tension between being labelled high risk and wanting to rebuild confidence in daily routines. Two points recurred: that recovery begins rather than ends when the operation is over, and that being continually reminded of patienthood was among the things a support tool should avoid. Monitoring, in this sense, was seen as something that could either support reintegration or reinforce fragility, depending on how it is designed (phase A, sessions 3 and 6).

### Proportionality and trust

When discussing digital systems, participants returned repeatedly to the issue of proportionality.

Alerts were acceptable if they were meaningful. Vague or frequent notifications were described as likely to increase tension, especially among those already inclined toward vigilance. Several participants noted that an alert should be clearly connected to an action: who to contact and how, within what time frame and why.

Participants rejected alerting on every abnormal value and the rendering of deviation as alarm. The condition they attached to any notification was that it be tied to an action: a warning that cannot say what should happen next was regarded as worse than no warning. In phase C this was developed into an explicit sequence, context first, then whether the finding is a pattern or a single occurrence, then an explanation to the patient, then re-measurement if indicated and only then a defined action, graded from self-monitoring through contact with the outpatient clinic to urgent assessment. Participants added that the recipient of any alert should be identified, that not every deviation should be routed to the hospital, and that no system should imply round-the-clock monitoring.

Accessibility was another recurring concern. Not everyone feels comfortable navigating complex digital platforms. Clear language, intuitive interfaces and minimal technical barriers were seen as essential conditions for participation.

The focus group convened for this purpose (phase B) identified what makes digital care usable and what makes it inaccessible. On the enabling side: a phone that is always to hand, clear graphical display, large type, plain explanation, a single place for information, the ability to choose, provision in languages other than Dutch, automatic recording of readings and the option to let a family member assist. On the obstructing side: multiple applications, passwords and national digital identity systems, medical vocabulary, small type, graphs whose meaning is unclear to the patient, frequent notifications, the fear of entering something incorrectly, dependence on a recent handset or a smartwatch and material available only in Dutch. Participants observed that if such a system becomes too demanding, the people most in need of support are the first to disengage.

Trust extended beyond data security. Participants wanted to understand who reviews the data, how often, and how it influences clinical decisions, and named the absence of that information as a reason not to take part. Knowing that information is actually integrated into care, rather than simply stored, appeared central to acceptance. Participants were equally clear that any such system should remain optional, should not displace the possibility of speaking to someone and should not be experienced as continuous observation (phase A, session 6).

### From conversation to structure

As the sessions progressed, participants gradually moved from describing experiences to outlining what would actually help in practice. The agenda did not emerge as a predefined framework, but as something that took shape while reflecting on concrete situations, moments of doubt, repeated blood pressure checks, uncertainty about exercise, fragmented advice. Several elements recurred consistently.

Participants wanted a structured way to record their symptoms. Almost all had a desire to make sense of the symptoms, rather than simply logging them. They asked for visual overviews of blood pressure over time, so that changes could be seen in context rather than judged on single values. Space for reporting well-being and anxiety was considered important, particularly when these influenced how physical sensations were interpreted.

Activity monitoring was viewed as useful when optional and contextual, something that could support confidence in returning to daily life rather than impose restrictions. Information tailored to life stage, including pregnancy and work participation, was described as helpful if it brought coherence to advice received across specialties. Finally, any form of alerting needed to be clearly linked to action and embedded in existing care pathways.

Across discussions, one principle remained consistent. Digital monitoring was expected to support dialogue with clinicians. Participants valued shared interpretation and clarity about who reviews the data and how it informs decisions. A system functioning in isolation from clinical relationships was not seen as desirable.

### Validation of the framework

In the phase D validation session, participants recognised all five domains and endorsed retaining them. They qualified two. Uncertainty and fear of recurrence was recognised as most pronounced after dissection and as changing with time since the event, with the observation that not everyone wants more monitoring and that monitoring can itself generate anxiety. Accessibility and proportionality was endorsed unconditionally, with the additions that use should remain the patient’s choice, that human care should be preserved and that not everyone should be asked to measure the same things. Participants also identified an omission within symptom interpretation: guidance on when to make contact was, in their experience, the element most often missing. No domain was rejected and none was added.

The relationship between the five patient-derived domains and the resulting components of the monitoring agenda is summarised in table 3 and in figure 1.

## Discussion

In this study, patients living with TAD described digital monitoring as something that could help them navigate daily life between hospital visits. Imaging follow-up provides structural information about the aorta, but it does not address the ongoing interpretive work patients describe doing at home. This observation resonates with prior work showing that recovery after aortic dissection extends beyond survival and involves ongoing challenges related to activity, sleep and quality of life, highlighting the need for supportive strategies between hospital encounters.^8 9 18^

Uncertainty was present across trajectories, although it manifested differently. Individuals after acute dissection often spoke about a changed relationship with their body. Sensations carried weight. Participants described hesitation: whether to seek care, whether to wait, whether a symptom was familiar or new. Among those in surveillance without prior acute events, uncertainty tended to cluster around imaging intervals, with tension building before scans and easing afterwards. These patterns suggest that digital tools, if used, would need to account for temporal rhythms of anxiety rather than treat risk perception as static. The prominence of fear and symptom vigilance aligns with reports of post-traumatic stress symptoms following acute type A aortic dissection, suggesting that monitoring strategies must balance reassurance with avoidance of anxiety amplification.^10 15^

Blood pressure monitoring provided a useful example of this complexity. Many participants already measure at home. Some experienced this as empowering, whereas others found it destabilising, particularly during stressful periods. Repeated measurements can escalate concern without clarifying meaning. Participants consistently asked for longitudinal representation, trends over time, contextual framing and guidance about what constitutes meaningful change. Their descriptions reflect an intuitive orientation toward trajectories rather than single cut-off values. While blood pressure control remains a cornerstone of medical management, these findings suggest that patient experience may be better supported by longitudinal interpretation rather than isolated measurements, particularly in populations undergoing long-term surveillance.^6^

Symptom interpretation was discussed in similar terms. Participants did not ask for automated triage. They asked for help in structuring information, to make sense of duration, intensity, associated features and to communicate more clearly with their clinicians. Digital documentation was seen as a possible bridge in that communication, provided it remained embedded within existing care relationships.

Life participation emerged as equally central. Return to exercise, work, caregiving and decisions around pregnancy were repeatedly raised. Women in particular described fragmented information regarding reproductive risk and long-term planning. These concerns sit alongside, rather than separate from, imaging surveillance. They shape daily decision-making and confidence. A digital agenda limited to physiological metrics would therefore capture only part of the lived experience of aortic disease. This broader framing of recovery is consistent with emerging literature emphasising functional recovery and participation as key outcomes in aortic disease survivors.^8 9^

Participants also articulated conditions under which digital monitoring would be acceptable. Systems must be interpretable, proportionate and clearly connected to clinical follow-up. Excessive or ambiguous alerts were viewed as likely to increase anxiety. Transparency about who reviews data and how it informs care was considered essential. The relational dimension of monitoring, knowing that data are meaningfully integrated into care, appeared more important than the volume of data collected. Practical considerations around remote care in thoracic aortopathy have previously been described, yet the present findings extend this work by highlighting patient-defined priorities for interpretation, proportionality and relational integration.^11^

Taken together, these findings suggest that digital monitoring in TAD should be approached as an extension of interpretive care. This contrasts with parameter-driven digital models that prioritise physiological thresholds over contextualised interpretation. The emphasis lies on contextualisation, longitudinal understanding and integration with clinical relationships. The agenda developed in this study reflects these priorities and offers a structured basis for prototype development.

### Strengths and limitations

This study set out a patient driven digital monitoring agenda for TAD through a multiphase qualitative co-creation design, shifting the from clinically defined parameters to what patients themselves consider worth following visits. To our knowledge, no previous study has undertaken co-creation across the stages of TAD. The multiple-phase design allowed findings to be developed and tested iteratively rather than generated in a single round, and purposive sampling across three clinical trajectories permitted comparison of post-dissection, post-surgical and surveillance experiences that are usually studied separately. Phase B was added specifically to recruit under-represented perspectives, coding was undertaken by two researchers who had not facilitated the sessions, and the resulting framework was returned to participants for validation.

Several limitations qualify these findings. The qualitative design carries no claim to statistical generalisability: the agenda describes priorities articulated by this population, not their distribution. Recruitment from a single tertiary referral centre is likely to over-represent patients who are engaged with specialist follow-up and willing to discuss their experience; those who disengage from surveillance, who have limited Dutch language proficiency, or who are managed in non-specialist settings may have different needs. Thematic sufficiency, as we use the term, is a statement about the adequacy of the material for developing an agenda within this group; it is not a claim that the domains would be exhaustive in another population or setting. Phase A material was moreover not collected as research data and phase A participants were not independent of the later phases, so the four phases cannot be treated as separate samples and recurrence across them is not independent corroboration.

Most consequentially, the sessions were not audio-recorded and no verbatim transcripts exist, so the analysis rests on participants’ own written material, on flip-chart records and on field notes. Nuance, hesitation and self-correction are therefore absent from the record; what was written down reflects the facilitator’s judgement at the time about what mattered; and material was pooled without individual attribution, so we cannot say whether a domain reflects many participants or a few, nor relate views to participant characteristics. The findings should be read as an account of what a group of patients collectively put forward rather than as a rendering of individual accounts.

Clinician perspectives were obtained only within the phase C workshop, and no clinician-only group was convened. This was deliberate: a parallel clinician-only strand risked reframing the agenda around clinically familiar parameters before patient priorities had been articulated. The cost is that we cannot identify where patient and clinician priorities diverge, which a comparative design would establish and which would bear directly on the design of escalation logic. Because the facilitators were clinicians at the treating centre, social desirability cannot be excluded. Finally, the study reports expressed needs rather than the performance of an implemented system: whether these components reduce uncertainty without amplifying vigilance requires prospective evaluation, and the agenda is a hypothesis-generating basis for prototype development rather than evidence of benefit.

## Conclusion

The patients with TAD who took part in this study described digital monitoring as a way to support interpretation and continuity in everyday life. The agenda they set out emphasises longitudinal patterns, structured symptom reflection, integration of life-course concerns and proportional escalation pathways. These elements provide direction for the next phase of digital surveillance development in aortic care.

## Supplementary material

10.1136/bmjopen-2026-121086 online supplemental file 1

10.1136/bmjopen-2026-121086 online supplemental file 2

10.1136/bmjopen-2026-121086 online supplemental file 3

## Data Availability

All data relevant to the study are included in the article or uploaded as supplementary information.
