# Supplementary material for: Co-creating a patient-driven digital monitoring agenda for thoracic aortic disease: a multiphase qualitative study
Source: BMJ Open. 2026 Sep 25;16(9):e121086. doi: 10.1136/bmjopen-2026-121086 (PMC13629912; doi:10.1136/bmjopen-2026-121086)
Supplement: Supplementary file 1 [file bmjopen-2026-121086.reviewer_comments.pdf]

PEER REVIEW HISTORY

BMJ Open publishes all reviews undertaken for accepted manuscripts. Reviewers are asked to complete a checklist review form and are provided with free text boxes to elaborate on their assessment. These free text comments are reproduced below.

ARTICLE DETAILS

Title (Provisional)

Co-creating a patient-driven digital monitoring agenda for thoracic aortic disease: a multi-phase qualitative study

Authors

Grewal, Nimrat; Bacour, Nora; Aouragh, Youness; Tirpan, Aytug U; Beukers, Anne M.; Grewal, Simran

---

VERSION 1 - REVIEW

**Reviewer**	**1**
**Name**	**Gholamzadeh, Marsa**
**Affiliation**	**Tehran University of Medical Sciences**
**Date**	**23-May-2026**
**COI**	**None**

This is a well-conducted, methodologically sound qualitative study that addresses an important gap in patient-centered digital health for thoracic aortic disease (TAD). The multi-phase co-creation approach is a strength, and the findings offer meaningful insights for future intervention development. However, several revisions are required to improve clarity, transparency, and alignment with reporting standards.

1- While the authors include a COREQ checklist, several items are incompletely reported or marked "not reported":

2- The authors state recruitment continued until "thematic sufficiency was reached" . Please clarify:

How was thematic sufficiency determined?

Were there any a priori stopping criteria?

3- The manuscript lacks a detailed participant characteristics table.

4- The authors note facilitators were aware of their clinical roles and reflected on potential influence. However, no specific examples of reflexivity are provided. Please add a brief paragraph in Methods or Discussion describing reflexivity:

5- The graphical abstract appears on Page 6 and again on Page 27 as an image. Consider removing duplication and ensuring the final version is placed appropriately for publication.

6- Line 3: "BackgroundBackground" – typo. Please correct to "Background."

7- The bulleted list of participant groups should be formatted consistently.

8- Specify how many patients vs. healthcare professionals participated in the co-design workshop (n=9 total, but breakdown not provided).

9- The discussion is appropriately contextualized with prior literature. However, the authors should acknowledge the absence of input from clinicians alone (without patients) as a potential limitation. Was clinician-only input sought to compare priorities?

10- Reference 14 is incomplete (Page 25, truncated at "Dutch National").

VERSION 1 - AUTHOR RESPONSE

Reviewer 1 — Dr Marsa Gholamzadeh, Tehran University of Medical Sciences

We thank Dr Gholamzadeh for a careful and generous reading, and for a set of comments that were specific enough to act on directly. We have addressed all ten points; the substantive ones (COREQ completeness, stopping criteria, participant characteristics, reflexivity and the absence of clinician-only input) have each produced new text in the manuscript, and the presentational ones have been corrected.

Comment 1

> *"While the authors include a COREQ checklist, several items are incompletely reported or marked "not reported"."*

Response:

We agree. Three items were marked "not reported" (23, 25 and 29) and several others were reported only thinly. All three are now reported in the manuscript, as set out in our response to the Executive Editor's first comment, and the checklist has been revised in full against the new manuscript with corrected page references. In addition, and in response to this reviewer's specific comments below, we have strengthened the reporting of item 8 (interviewer characteristics and assumptions) through a new reflexivity subsection, item 16 (description of the sample) through a new table 1, item 17 (interview guide and pilot testing) through a new account of guide development and a supplemental file containing the guide, and item 22 (data saturation) through an explicit statement of a priori stopping criteria.

We have also added, in the Discussion, an acknowledgement of the limits that the phase A material imposes on what can be reported, since this underlies part of the incompleteness the reviewer identified.

Manuscript change (p.25, l.726 — Discussion, Strengths and limitations — nature of the record). New / amended text reads:

"Most consequentially, the sessions were not audio-recorded and no verbatim transcripts exist, so the analysis rests on participants' own written material, on flip-chart records and on field notes. Nuance, hesitation and self-correction are therefore absent from the record; what was written down reflects the facilitator's judgement at the time about what mattered; and material was pooled without individual attribution, so we cannot say whether a domain reflects many participants or a few, nor relate views to participant characteristics. The findings should be read as an account of what a group of patients collectively put forward rather than as a rendering of individual accounts."

COREQ item	Previously	Now	Where
23. Transcripts returned to participants	Not reported	Reported: no transcripts were produced, so none were returned. The two forms participant checking did take are described, together with the acknowledgement that neither is equivalent to returning a transcript.	Methods, Phase D
25. Description of the coding tree	Not reported	Reported: no codebook or coding tree was used or retained, with the reasoning set out and the two Braun and Clarke methodological references added (now references 14 and 15).	Methods, Data analysis
29. Quotations presented	Not reported	Reported: no verbatim record exists, so no quotations are presented. In their place, the complete output of every elicitation exercise is reproduced under the prompt that generated it as online supplemental file 3, and each finding is attributed to the phase and session that generated it.	Methods, Data analysis; supplemental file 3
8. Interviewer characteristics and assumptions	Reported thinly (one sentence)	Expanded into a dedicated reflexivity subsection with specific issues, what was done about each, and an analytic decision that changed as a result.	Methods, Research team and reflexivity
16. Description of the sample	Reported thinly	New table 1 (participant characteristics, aggregate only) and new table 2 (all ten sessions across the four phases, with prompt and form of record).	Methods, Participants and sampling; Data collection
17. Interview guide	Reported thinly	The 35-question guide is supplied in full, in Dutch with English translation, as online supplemental file 2, with a description of its structure and of how it was used.	Methods, Phase B; supplemental file 2
19. Audio/visual recording	Reported as page 8–9	Corrected: sessions were not audio-recorded. The	Methods, Data collection

		original entry was inaccurate.	
22. Data saturation	Reported as page 8	Replaced by an explicit account of how thematic sufficiency was judged, in place of the bare assertion that it 'was reached'.	Methods, Participants and sampling

Comment 2

> *"The authors state recruitment continued until "thematic sufficiency was reached". Please clarify: How was thematic sufficiency determined? Were there any a priori stopping criteria?"*

Response:

The original sentence asserted sufficiency without saying how it was judged, which was not adequate. We have added an explicit account of how the judgement was made and of the criteria set before data collection began, and we have explained why we use the term thematic sufficiency rather than saturation.

Manuscript change (p.8, l.263 — Methods, Participants and sampling). New / amended text reads:

> "Rather than fixing a sample size in advance, we specified stopping criteria before data collection in phase B and framed these in terms of thematic sufficiency, understood as information power, rather than as data saturation in the inductive-inferential sense. Three criteria were defined a priori: (i) that all three clinical trajectories were represented and that the perspectives identified as underrepresented in phase A had been purposively recruited; (ii) that two consecutive sessions yielded no new codes that changed the content or the boundaries of the candidate domains; and (iii) that the research team judged the material sufficient to allow each domain to be translated into concrete monitoring components in phase C. These criteria were reviewed by the research team after every session. All three were met after the second focus group in phase B, and recruitment was closed at that point. We use the term thematic sufficiency in preference to saturation because the analytic aim was to develop an agenda that was adequately informed across trajectories, not to demonstrate that no further themes could ever be identified."

The original sentence "Recruitment continued until thematic sufficiency was reached across groups." has been deleted and replaced by the passage above. COREQ item 22 has been updated to point to it.

Comment 3

> *"The manuscript lacks a detailed participant characteristics table. [Editors' note: please ensure that the information reported cannot lead to the identification of participants — for example, data can be presented in summary form only rather than details of individual participants. If details of individual participants are reported in a table or elsewhere, we allow no direct identifiers and maximum of two indirect identifiers (with, for example, numerical data such as age presented as ranges rather than absolute values), in order to avoid individuals being identifiable.]"*

Response:

A participant characteristics table has been added as table 1. We have followed the Editors' guidance closely, and we note that it constrains the form the table can take: because our participants are drawn from a rare disease population attending a single national referral centre, a row-per-participant table would in our judgement be identifying even without direct identifiers. Table 1 therefore reports characteristics in aggregate by study phase only. There are no individual-level rows, age is given as a median with a range rather than as absolute values, and time since the index event is banded rather

than exact. We have also refrained from cross-tabulating characteristics (for example sex by trajectory by age band), since combining cells in a group of nine would reintroduce identifiability.

Manuscript change (p.8, l.275 — Methods, Participants and sampling — lead-in to table 1). New / amended text reads:

> "Participant characteristics are summarised by study phase in table 1. In line with the journal's policy on participant privacy, characteristics are reported in aggregate only; no individual-level rows are presented and age is given as a range."

Manuscript change (p.30, l.826 — New table 1). New / amended text reads:

> "New table 1, "Characteristics of participants by study phase", reproduced below."

Characteristic	Phase A (n=45)	Phase B (n=13)	Phase C (n=9)	Phase D (n=9)
Age, years — median (range)	56 (45–65)	54 (45–65)	57 (46–64)	56 (47–65)
Women — n	20	9	4	5
Clinical trajectory — n				
Acute aortic dissection, surgically managed	15	4	2	3
Elective aortic surgery (± valve intervention)	15	4	2	3
Aneurysm / bicuspid aortic valve under surveillance	15	5	2	3
Time since diagnosis or index event — n				
<2 years	18	5	2	3
2–5 years	18	5	3	4
>5 years	9	3	1	2
Healthcare professionals — n	0	0	3	0
Sessions	6 engagement sessions	2 focus groups	1 co-design workshop	1 online validation session

We have additionally added table 2, which sets out how each of the five patient-derived domains maps onto the components of the monitoring agenda and the design conditions participants attached to them. This was not requested, but in preparing the response we found that the step from domains to agenda was asserted in prose rather than shown, and the table makes that translation auditable.

Patient-derived domain	Core patient concern	Component of the digital monitoring agenda	Design conditions specified by participants
1. Managing uncertainty and fear of recurrence	Uncertainty is continuous after dissection and cyclical around imaging in surveillance; reassurance is time-limited	Patient-reported outcomes covering wellbeing and anxiety, timed to the surveillance cycle	Must reduce ambiguity rather than generate further data; optional rather than obligatory completion
2. Interpreting symptoms and bodily signals	Difficulty judging whether a sensation is clinically relevant, and difficulty describing it to a clinician	Symptom contextualisation tool structuring duration, progression, associated features and comparison with previous episodes	Structured reflection, not automated triage; output intended to support the consultation, not replace it

3. Longitudinal blood pressure patterns	Single readings drive repeated measurement and anxiety; participants reason in trends, not thresholds	Longitudinal blood pressure trend visualisation with contextual framing	Trend display prioritised over isolated values; consistent guidance on measurement frequency and on what constitutes meaningful change
4. Life participation (activity, pregnancy, work)	Generic advice does not answer 'what is safe for me'; reproductive and occupational guidance arrives fragmented across specialties	Contextual activity feedback and educational decision-support modules tailored to life stage	Optional and confidence-building rather than restrictive; coherent across specialties
5. Accessibility and proportionality	Frequent or vague alerts increase tension; access barriers exclude; storage without use erodes trust	Transparent escalation pathways embedded in existing care pathways	Every alert linked to a named action, contact and timeframe; plain language and low technical threshold; explicit statement of who reviews data and how often

Comment 4

"The authors note facilitators were aware of their clinical roles and reflected on potential influence. However, no specific examples of reflexivity are provided. Please add a brief paragraph in Methods or Discussion describing reflexivity."

Response:

We accept that the original sentence claimed reflexivity without demonstrating it. The Methods now contain a dedicated subsection, "Research team and reflexivity", which sets out three specific issues that arose, what was done about each, and — in the third case — an analytic decision that changed as a result. We have also added a corresponding limitation to the Discussion acknowledging that these mitigations do not eliminate the risk of social desirability.

Manuscript change (p.6, l.227 — Methods — new subheading). New / amended text reads:

"Research team and reflexivity"

Manuscript change (p.6, l.235-255 — Methods, Research team and reflexivity). New / amended text reads:

"The facilitators were aware that their clinical roles could shape what participants felt able to say, and reflexivity was addressed prospectively rather than only during analysis. Three issues were identified and documented in a reflexive log kept throughout the study. First, because NG is a cardiothoracic surgeon at the centre where participants receive their care, there was a risk that participants would withhold criticism of the follow-up they had received; each session therefore opened with an explicit statement that the purpose was to inform future care, that nothing said would affect the participant's own treatment, and that critical views were particularly welcome, and NG did not facilitate discussion with participants for whom she was the operating surgeon. Second, both facilitators are clinicians with a prior interest in remote monitoring, which risked steering discussion toward the feasibility of technology rather than toward whether monitoring was wanted at all; this became visible when early coding produced a disproportionate number of feature-oriented codes, and the topic guide was subsequently reordered so that questions about experience and unmet need preceded any mention of digital tools. Third, the initial coding was performed by the research team who had not facilitated the sessions, and their readings were compared with those of the facilitators specifically to surface interpretations that reflected clinical framing rather than participants' accounts; the decision to retain 'proportionality and trust' as a domain in its own right, rather than treating it as a design requirement subordinate to the clinical domains, followed from one such discussion."

The original sentence "The facilitators were aware of their clinical roles and reflected on potential influence during analytic discussions within the research team." has been deleted and replaced by the passage above.

Comment 5

> *"The graphical abstract appears on Page 6 and again on Page 27 as an image. Consider removing duplication and ensuring the final version is placed appropriately for publication."*

Response:

Corrected. The duplication arose because the image was both embedded in the manuscript file and uploaded separately. Following the Research Editor's instruction (comment 7), the graphical abstract has been converted into figure 1: the embedded copy and the heading "Graphical abstract" have been removed from the main manuscript Word file, the image is supplied once as a separate 'Figure' file, and only the legend appears in the Word file, after the reference list. Figure 1 is cited at the end of the Introduction and in the Methods. Two typographical errors inside the image have also been corrected in the resupplied version ("Patiënt-Reported Outcomes" to "Patient-Reported Outcomes" and "Tranparant Escalation Pathways" to "Transparent Escalation Pathways".

Manuscript change (p.35, l.881-888 — Figure legend, after the reference list; the image itself is supplied as a separate Figure file). New / amended text reads:

> "Figure 1 Overview of the multi-phase co-creation process, the resulting patient-derived domains and the patient-driven digital monitoring agenda for thoracic aortic disease. The left-hand column shows the three clinical trajectories from which participants were purposively sampled (surgically managed acute aortic dissection, elective aortic surgery, and thoracic aneurysm under imaging surveillance). The second column shows the four sequential study phases with the number of participants in each. The third column shows the five patient-derived domains identified through reflexive thematic analysis. The right-hand column shows the six components of the resulting digital monitoring agenda. Created with BioRender.com."

Comment 6

> *"Line 3: "BackgroundBackground" – typo. Please correct to "Background.""*

Response:

Thank you for catching this. The duplication was introduced when the abstract text was merged with the heading in the submission file. It no longer arises: following the Research Editor's comment 2, the abstract has been restructured under the BMJ Open subheadings for Original Research and the heading "Background" has been replaced by "Objectives". We have checked the remaining headings in the revised file individually for the same fault.

Manuscript change (p.2, l.30 — Abstract — first subheading (replaces the duplicated "Background" heading)). New / amended text reads:

> "Objectives"

Comment 7

> *"The bulleted list of participant groups should be formatted consistently."*

Response:

Corrected. Both lists in the Participants and sampling subsection are now formatted identically: the same bullet style, sentence case throughout, a semicolon after each item and a full stop after the last, and consistent grammatical form across items. In the first list we have also aligned the wording with

the abstract, so that the three trajectories are described identically in both places, and we have expanded “±” to “with or without”.

Manuscript change (p.7, l.260-262 — Methods, Participants and sampling — first list). New / amended text reads:

> surgically managed acute aortic dissection;
>
> elective aortic surgery for aneurysm with or without valve intervention;
>
> thoracic aneurysm or bicuspid aortic valve under imaging surveillance.

Manuscript change (p.7, l.266-267 — Methods, Participants and sampling — second list). New / amended text reads:

> women across reproductive and post-reproductive life stages;
>
> participants with varying digital literacy and socioeconomic backgrounds.

Comment 8

> *“Specify how many patients vs. healthcare professionals participated in the co-design workshop (n=9 total, but breakdown not provided).”*

Response:

The breakdown is now stated explicitly at the start of the Phase C description rather than at the end of the paragraph, where it was easy to miss: nine participants, comprising six patients purposively selected to represent all three clinical trajectories, and three healthcare professionals. We are grateful for this comment for a second reason: checking it showed that the original text named four disciplines (cardiothoracic surgeon, cardiologist, nurse specialist and clinical geneticist) for three people, which cannot both be right. The revised text names the three who were present. The breakdown now also appears in the abstract under Participants and in table 1, and phase D is recorded as comprising nine patient participants with no healthcare professionals.

Manuscript change (p.10, l.358-364 — Methods, Phase C). New / amended text reads:

> “A structured workshop brought together nine participants: six patients, purposively selected to represent all three clinical trajectories, and three healthcare professionals. Using facilitated small-group exercises, participants translated identified themes into potential digital features, escalation logic, and governance principles.”

Comment 9

> *“The discussion is appropriately contextualized with prior literature. However, the authors should acknowledge the absence of input from clinicians alone (without patients) as a potential limitation. Was clinician-only input sought to compare priorities?”*

Response:

To answer the reviewer's direct question first: no, clinician-only input was not sought. This was a deliberate design decision rather than an oversight, but the reviewer is right that its consequence was not acknowledged, and we have now stated both the reasoning and the cost. Clinicians participated only within the mixed-stakeholder workshop in phase C, by which point the five domains had already been established from phases A and B. We have added a limitation setting out what this means for the interpretation of the agenda, including the point that we cannot identify where patient and clinician priorities diverge, and we have proposed the comparative design that would answer the question. This limitation is also reflected in bullet 5 of the Strengths and limitations of this study section.

Manuscript change (p.25, l.733-742— Discussion, Strengths and limitations). New / amended text reads:

"Clinician perspectives were obtained only within the phase C workshop, and no clinician-only group was convened. This was deliberate: a parallel clinician-only strand risked reframing the agenda around clinically familiar parameters before patient priorities had been articulated. The cost is that we cannot identify where patient and clinician priorities diverge, which a comparative design would establish and which would bear directly on the design of escalation logic. Because the facilitators were clinicians at the treating centre, social desirability cannot be excluded. Finally, the study reports expressed needs rather than the performance of an implemented system: whether these components reduce uncertainty without amplifying vigilance requires prospective evaluation, and the agenda is a hypothesis-generating basis for prototype development rather than evidence of benefit."

Manuscript change (p.3, l.137-139 — Strengths and limitations of this study, bullet 5). New / amended text reads:

"All participants were recruited from a single tertiary referral centre and no clinician-only group was convened, so the findings may over-represent patients who are actively engaged in specialist follow-up and do not permit a formal comparison of patient-derived with clinician-derived priorities."

Comment 10

"Reference 14 is incomplete (Page 25, truncated at "Dutch National")."

Response:

We have checked this against the source file. The reference is complete; the apparent truncation is an artefact of the ScholarOne proof, in which the entry breaks across the foot of one page and continues at the top of the next ("Aortic Dissection Advocacy Group. Journal of clinical medicine 2023;12(20)…"). No text was missing.

One point of navigation. Two methodological references (Braun and Clarke, 2019 and 2021) have been added in response to the comments on reflexive thematic analysis, and they have been placed after the COREQ reference. The reference the reviewer refers to as number 14 is therefore numbered 16 in the revised manuscript, and the reference list now contains 17 entries rather than 15.

Manuscript change (p.29, l.815 — Reference 16 in the revised numbering (reference 14 in the original), verified complete). New / amended text reads:

"16. Breel JS, de Klerk ES, Strypet M, et al. What Really Matters to Survivors of Acute Type A Aortic Dissection-A Survey of Patient-Reported Outcomes in the Dutch National Aortic Dissection Advocacy Group. Journal of clinical medicine 2023;12(20) doi: 10.3390/jcm12206584 [published Online First: 20231018]"

Manuscript change (p.29, l.810 — References 14 and 15, newly added). New / amended text reads:

14. Braun V, Clarke V. Reflecting on reflexive thematic analysis. Qualitative Research in Sport, Exercise and Health 2019;11(4):589-97.

15. Braun V, Clarke V. One size fits all? What counts as quality practice in (reflexive) thematic analysis? Qualitative Research in Psychology 2021;18(3):328-52. doi: 10.1080/14780887.2020.1769238

Summary of changes

For convenience, the table below lists every change made in this revision and where it can be found in the marked copy.

Change	Location	Prompted by
Title in sentence case	p.1, l.1	RE1

Abstract restructured to BMJ Open subheadings	p.2, l.30	RE2
New: Strengths and limitations of this study	p.3, l.122-139	RE3, RE4
New: Research team and reflexivity subsection	p.6, l.235-255	R1.4
New: account of thematic sufficiency	p.8, l.263	R1.2
New: table 1, participant characteristics	p.30, l.826	R1.3
New: description of the record	p.9, l.317-325	EE1, EE2
New: table 2, all sessions with prompt and record	p.32, l.837	EE2
New: findings attributed to phase and session	p.14, l.444	EE2, R1.1
New: reporting convention stated	p.12, l.430-449	EE2
New: COREQ item 23 reported	p.11, l.392-398	EE1
New: COREQ item 25 reported	p.11, l.411-426	EE1
New: table 3, domains mapped to agenda	p.34, l.846	R1.3
Expanded: limitations	p.25, l.720-722	RE4, R1.9
New: clinician-only input as a limitation	p.25, l.733-742	R1.9
New: Data availability statement	p.27, l.774-786	RE5
Graphical abstract converted to figure 1	p.4, l.180; legend p.35, 881-888	RE7, R1.5
Phase C composition specified	p.10, l.358-264	R1.8
Bullet lists made consistent	p.7, l.260-267	R1.7
Braun and Clarke added as references 14 and 15	p.29, l.810	R1.10, EE1

We thank the Editors and Reviewer 1 again for their engagement with the manuscript. We believe the paper is considerably stronger for this revision, particularly in the reporting of its methods and in the evidential grounding of the Results.

VERSION 2 - REVIEW

Reviewer 1

Name Gholamzadeh, Marsa

Affiliation Tehran University of Medical Sciences

Date 09-Sep-2026

COI

While the authors have improved the description of stopping criteria, there remains a tension between claiming "thematic sufficiency" and the limited scope of recruitment (single centre, self-selecting participants). The manuscript states that findings "describe priorities articulated by this population, not their distribution" (p.25), which is appropriate, but some language elsewhere implies broader applicability.

The authors are transparent that Phase A materials were not originally designed as research data and lack audio-recording. The account of how these materials were used is now much clearer. However, it remains somewhat unclear how findings from Phase A were distinguished from those emerging in Phase B-D, given that Phase A participants were not independent and the prompts differed.

The abstract's Methods section (p.2) has some awkward phrasing and redundancy from the revision process.

Reference 4 (Nappi et al., 2023) is listed without full page numbers.

The figure legend states "Created with BioRender.com" but the image quality in the PDF is moderate; some text appears slightly compressed.

The COREQ checklist (supplemental file 1) has page numbers referencing the revised manuscript but the page references may shift during final typesetting.

This is a well-executed qualitative study that makes a meaningful contribution to patient-centred digital health research in aortic disease. The authors have been exemplary in responding to previous feedback. The remaining suggestions are minor clarifications that will further strengthen an already strong manuscript.

VERSION 2 - AUTHOR RESPONSE

Reviewer 1 — Dr Marsa Gholamzadeh, Tehran University of Medical Sciences

We thank the reviewer for a second reading that is as precise as the first. The six points are answered in turn.

Comment 1

> *"While the authors have improved the description of stopping criteria, there remains a tension between claiming "thematic sufficiency" and the limited scope of recruitment (single centre, self-selecting participants). The manuscript states that findings "describe priorities articulated by this population, not their distribution" (p.25), which is appropriate, but some language elsewhere implies broader applicability."*

Response:

The tension is real and we have resolved it in the direction the reviewer indicates. Rather than defend the term, we now say what it does and does not claim, in the same paragraph in which it is used, and we have brought the two places where the text still generalised into line with the sentence the reviewer quotes.

First, the limitations paragraph now states the scope of the sufficiency claim explicitly.

Manuscript change (p.24, l.573 — Discussion, Strengths and limitations — added to the first limitations paragraph). New / amended text reads:

> "Thematic sufficiency, as we use the term, is a statement about the adequacy of the material for developing an agenda within this group; it is not a claim that the domains would be exhaustive in another population or setting."

Second, the Conclusion said “Patients with TAD describe digital monitoring as…”, which reads as a claim about patients with the condition in general. It is now tied to the participants studied.

Manuscript change (p.25, l.674 — Conclusion). New / amended text reads:

> “The patients with TAD who took part in this study described digital monitoring as a way to support interpretation and continuity in everyday life. The agenda they set out emphasises longitudinal patterns, structured symptom reflection, integration of life-course concerns, and proportional escalation pathways.”

Third, the abstract Conclusions carried the same implication and now carry the same qualification.

Manuscript change (p.3, l.66 — Abstract, Conclusions). New / amended text reads:

> “Structured patient engagement and iterative co-design produced a patient-driven digital monitoring agenda for thoracic aortic disease integrating biomedical and psychosocial dimensions, describing priorities articulated by these participants rather than their distribution, and providing a foundation for developing and evaluating hybrid digital surveillance models in aortic care.”

We re-read the Results and Discussion for other unqualified generalisations and found none that required change; the sentence the reviewer quotes has been kept as it stands.

Comment 2

> *“The authors are transparent that Phase A materials were not originally designed as research data and lack audio-recording. The account of how these materials were used is now much clearer. However, it remains somewhat unclear how findings from Phase A were distinguished from those emerging in Phase B-D, given that Phase A participants were not independent and the prompts differed.”*

Response:

This was implicit in the text and should not have been. The two features the reviewer names, non-independence of participants and differing prompts, are exactly why phase A material was treated as a starting point rather than as corroboration, and the Methods now say so and set out what follows from it. A new paragraph has been added at the end of the Data analysis subsection.

Manuscript change (p.12, l.290 — Methods, Data analysis — new paragraph). New / amended text reads:

> “Phase A material was generated before this study was designed, under prompts that differed from those used later, and some phase A participants also took part in later phases. It was therefore used to identify candidate domains and to establish which perspectives were underrepresented, which is what phase B was then recruited to address, rather than as an independent source of corroboration. Every finding reported in the Results is attributed to the phase and session it rests on, and table 2 states the prompt used in each session, so that the material behind each claim can be identified. Because participants overlapped between phases, recurrence of a domain across phases is reported as recurrence within one connected group rather than as replication in independent samples.”

The consequence is also stated as a limitation, so that a reader who goes to the Discussion first does not have to reconstruct it.

Manuscript change (p.25, l.652 — Discussion, Strengths and limitations). New / amended text reads:

> “Phase A material was moreover not collected as research data and phase A participants were not independent of the later phases, so the four phases cannot be treated as separate samples and recurrence across them is not independent corroboration.”

Table 2, which gives the prompt and the form of the record for every session in every phase, and the attribution of each finding in the Results to the phase and session it rests on, are the practical means by which a reader can separate the two. Both were added in the previous revision and are unchanged.

Comment 3

> *"The abstract's Methods section (p.2) has some awkward phrasing and redundancy from the revision process."*

Response:

Corrected. The Design entry said "Multi-phase … with four sequential phases", and described phase A as a "secondary thematic analysis" two lines before stating that "Analysis used reflexive thematic analysis". Both redundancies are gone.

Manuscript change (p.2, l.31 — Abstract, Design). New / amended text reads:

> "Design Qualitative co-creation study in four sequential phases: secondary analysis of existing patient engagement material (A), targeted focus groups addressing underrepresented perspectives (B), a mixed-stakeholder co-design workshop (C), and a member-check validation session (D). Analysis used reflexive thematic analysis and framework synthesis. Reported following COREQ."

Two further sentences in the abstract have been tightened, in Participants and in Results, so that the additions made in answer to the comments above keep the abstract within the 300-word limit. It now runs to 299 words.

Manuscript change (p.2, l.38 — Abstract, Participants). New / amended text reads:

> "Phase A, 45 participants in six sessions; phase B, 13 in two focus groups; phase C, nine (six patients, three healthcare professionals); phase D, nine from earlier phases."

Manuscript change (p.2, l.49 — Abstract, Results — final sentence). New / amended text reads:

> "Participants framed monitoring as an adjunct to imaging surveillance, not a replacement, emphasising interpretability and restrained alerting."

Comment 4

> *"Reference 4 (Nappi et al., 2023) is listed without full page numbers."*

Response:

The page range was present but abbreviated in the journal's numbering style (6604-22). It is now given in full and the DOI has been added.

Manuscript change (p.28, l.632 — Reference 4). New / amended text reads:

> "4. Nappi F, Gambardella I, Singh SSA, et al. Survival following acute type A aortic dissection: a multicenter study. Journal of thoracic disease 2023;15(12):6604-6622. doi: 10.21037/jtd-23-1137"

Because the same abbreviation appeared elsewhere in the list, we have expanded every abbreviated end page (references 5, 6, 7, 13, 14, 15 and 17) so that the list is internally consistent, and added the missing DOI to reference 14. We also corrected two errors of our own in reference 6: the author name was inverted ("Mestral Cd") and the DOI prefix was duplicated.

Manuscript change (p.28, l.641 — Reference 6). New / amended text reads:

> "6. An KR, de Mestral C, Tam DY, et al. Surveillance Imaging Following Acute Type A Aortic Dissection. JACC 2021;78(19):1863-1871. doi: 10.1016/j.jacc.2021.08.058"

Comment 5

“The figure legend states "Created with BioRender.com" but the image quality in the PDF is moderate; some text appears slightly compressed.”

Response:

Figure 1 has been re-exported from BioRender at publication resolution and uploaded as a separate high-resolution image file rather than embedded, which is what degraded it in the previous proof. We took the opportunity to correct a spelling error in the last box of the figure, which read “Transparant Escalation Pathways” and now reads “Transparent Escalation Pathways”. The legend is unchanged.

Comment 6

“The COREQ checklist (supplemental file 1) has page numbers referencing the revised manuscript but the page references may shift during final typesetting. [NOTE FROM THE EDITORS: This is correct - while not a requirements, the authors may wish to consider revising the checklist to refer to sections and subsections of the manuscript, rather than page/line numbers]”

Response:

We have taken the suggestion. Every entry in online supplemental file 1 now refers to a section and subsection of the manuscript rather than to a page, for example “Methods — Data collection, phase B: targeted focus groups” in place of “p. 10”. The three items that were previously marked “not reported” (23, 25 and 29) keep the short statement of why, alongside the section in which it is given. The checklist is therefore unaffected by typesetting.

Closing remark

“This is a well-executed qualitative study that makes a meaningful contribution to patient-centred digital health research in aortic disease. The authors have been exemplary in responding to previous feedback. The remaining suggestions are minor clarifications that will further strengthen an already strong manuscript.”

We thank the reviewer. The two substantive points, the scope of the sufficiency claim and the standing of phase A material, have improved the paper in the places where it was least explicit about its own limits.

We thank the Editors and Reviewer 1 for their continued engagement with the manuscript.
