# Supplementary material for: Co-creating a patient-driven digital monitoring agenda for thoracic aortic disease: a multiphase qualitative study
Source: BMJ Open. 2026 Sep 25;16(9):e121086. doi: 10.1136/bmjopen-2026-121086 (PMC13629912; doi:10.1136/bmjopen-2026-121086)
Supplement: Supplementary file 2 [file bmjopen-16-9-s001.docx]

**Online supplemental file 1: COREQ checklist**

**Consolidated criteria for reporting qualitative studies (COREQ): 32-item checklist**

*Co-creating a patient-driven digital monitoring agenda for thoracic aortic disease: a multi-phase qualitative study*

Developed from: Tong A, Sainsbury P, Craig J. Consolidated criteria for reporting qualitative research (COREQ): a 32-item checklist for interviews and focus groups. International Journal for Quality in Health Care 2007;19(6):349–357.

References are given by section and subsection of the manuscript rather than by page number, so that they remain correct after typesetting. Three items previously marked “not reported” (23, 25 and 29) are reported in the Methods, with the reason in each case.

| Item No | **Guide Questions/Description** | **Reported in section** |
| --- | --- | --- |
| **Domain 1: Research team and reflexivity** |  |  |
| **Personal Characteristics** |  |  |
| 1. Interviewer/facilitator | Which author/s conducted the interview or focus group? | Methods — Research team and reflexivity |
| 2. Credentials | What were the researcher’s credentials? E.g., PhD, MD | Methods — Research team and reflexivity |
| 3. Occupation | What was their occupation at the time of the study? | Methods — Research team and reflexivity |
| 4. Gender | Was the researcher male or female? | Methods — Research team and reflexivity |
| 5. Experience and training | What experience or training did the researcher have? | Methods — Research team and reflexivity |
| **Relationship with participants** |  |  |
| 6. Relationship established | Was a relationship established prior to study commencement? | Methods — Research team and reflexivity |
| 7. Participant knowledge of the interviewer | What did the participants know about the researcher? | Methods — Research team and reflexivity |
| 8. Interviewer characteristics | What characteristics were reported about the interviewer/facilitator? e.g. bias, assumptions, reasons and interests in the research topic | Methods — Research team and reflexivity |
| **Domain 2: Study design** |  |  |
| **Theoretical framework** |  |  |
| 9. Methodological orientation and theory | What methodological orientation was stated to underpin the study? | Methods — Data analysis |
| **Participant selection** |  |  |
| 10. Sampling | How were participants selected? | Methods — Participants and sampling |
| 11. Method of approach | How were participants approached? | Methods — Participants and sampling |
| 12. Sample size | How many participants were in the study? | Methods — Participants and sampling; table 1 |
| 13. Non-participation | How many people refused to participate or dropped out? Reasons? | Methods — Participants and sampling |
| **Setting** |  |  |
| 14. Setting of data collection | Where was the data collected? | Methods — Data collection, phase B: targeted focus groups |
| 15. Presence of nonparticipants | Was anyone else present besides the participants and researchers? | Methods — Data collection, phase B: targeted focus groups |
| 16. Description of sample | What are the important characteristics of the sample? | Methods — Participants and sampling; table 1 |
| **Data collection** |  |  |
| 17. Interview guide | Were questions, prompts, and guides provided by the authors? Was it pilot tested? | Methods — Data collection, phase B: targeted focus groups; online supplemental file 2 |
| 18. Repeat interviews | Were repeat interviews carried out? If yes, how many? | Not applicable — no interviews were conducted; the study used group sessions (Methods — Data collection) |
| 19. Audio/visual recording | Did the research use audio or visual recording to collect the data? | Methods — Data collection, phase A: no; sessions were not audio-recorded |
| 20. Field notes | Were field notes made during and/or after the interview or focus group? | Methods — Data collection, phase A |
| 21. Duration | What was the duration of the interviews or focus group? | Methods — Data collection, phase B: targeted focus groups |
| 22. Data saturation | Was data saturation discussed? | Methods — Participants and sampling (stopping criteria; thematic sufficiency rather than saturation) |
| 23. Transcripts returned | Were transcripts returned to participants for comment and/or correction? | Methods — Data collection, phase D: member-check validation: no transcripts were produced |
| **Domain 3: Analysis and findings** |  |  |
| **Data analysis** |  |  |
| 24. Number of data coders | How many data coders coded the data? | Methods — Data analysis |
| 25. Description of the coding tree | Did the authors provide a description of the coding tree? | Methods — Data analysis: no coding tree was retained; table 3 sets out the relation between domains and agenda components |
| 26. Derivation of themes | Were themes identified in advance or derived from the data? | Methods — Data analysis |
| 27. Software | What software, if applicable, was used to manage the data? | Methods — Data analysis |
| 28. Participant checking | Did participants provide feedback on the findings? | Methods — Data collection, phase D; Results — Validation of the framework |
| **Reporting** |  |  |
| 29. Quotations presented | Were participant quotations presented to illustrate the themes/findings? Was each quotation identified? | Methods — Data analysis (reporting conventions): no verbatim record exists; the complete exercise output is in online supplemental file 3 |
| 30. Data and findings consistent | Was there consistency between the data presented and the findings? | Results (all subsections) |
| 31. Clarity of major themes | Were major themes clearly presented in the findings? | Results (all subsections) |
| 32. Clarity of minor themes | Is there a description of diverse cases or a discussion of minor themes? | Results (all subsections) |
