# Supplementary material for: Co-creating a patient-driven digital monitoring agenda for thoracic aortic disease: a multiphase qualitative study
Source: BMJ Open. 2026 Sep 25;16(9):e121086. doi: 10.1136/bmjopen-2026-121086 (PMC13629912; doi:10.1136/bmjopen-2026-121086)
Supplement: Supplementary file 3 [file bmjopen-16-9-s002.docx]

**Online supplemental file 2**

**Topic guide used in the patient sessions**

*Co-creating a patient-driven digital monitoring agenda for thoracic aortic disease: a multi-phase qualitative study*

**Development of the guide**

The guide was drafted jointly by NG and SG and was revised by the full research team before use. Three sources fed into it: the questions patients most often raise in aortic follow-up consultations at our centre; the themes identified in the team's earlier work on life impact, activity, sleep and quality of life after acute aortic dissection; and the uncertainty, symptom appraisal and psychological burden described in the wider aortopathy literature. Questions were written in lay Dutch, grouped into the eight areas below, and ordered so that experience and unmet need were covered before digital tools were introduced. The guide was not pilot tested in a separate session outside the study; the first phase B focus group served as its first use, no changes to wording or order were required afterwards, and the same guide was used in both phase B focus groups. The guide was used flexibly rather than administered question by question: not every question was put in every session, the order followed the discussion, and facilitators followed up on what participants raised. Sessions in phase A used a subset of these questions together with the written exercises listed in table 2 of the main manuscript.

**Questions (original Dutch)**

**Opening — leven met thoracale aortaziekte**

1. Als u in een paar woorden zou moeten beschrijven hoe het is om met uw aortaziekte te leven, welke woorden komen dan bij u op?

2. Wat is er voor u veranderd sinds de diagnose, dissectie of operatie?

3. Zijn er dingen waar u nu anders over denkt of anders mee omgaat dan vóór uw diagnose of behandeling?

**Onzekerheid en klachten**

4. Op welke momenten merkt u in het dagelijks leven het meest dat u een aandoening van de aorta heeft?

5. Wat gebeurt er wanneer u pijn, een vreemd gevoel of andere lichamelijke klachten ervaart?

6. Hoe bepaalt u of een klacht iets is om u zorgen over te maken?

7. Wanneer besluit u contact op te nemen met het ziekenhuis of een arts? Wat maakt die beslissing soms moeilijk?

8. Zijn er momenten waarop u zich juist gerustgesteld voelt?

**Controles en scans**

9. Hoe ervaart u de periode tussen twee controles of scans?

10. Verandert dat wanneer een nieuwe scan of controle dichterbij komt?

11. Wat geeft u na een controle of scan vertrouwen of geruststelling?

12. Welke vragen of onzekerheden blijven na een controle soms bestaan?

**Bloeddruk**

13. Meet u thuis uw bloeddruk? Hoe ervaart u dat?

14. Wanneer helpt het meten van uw bloeddruk u?

15. Zijn er ook situaties waarin meten juist onrust veroorzaakt?

16. Wat doet u wanneer u een onverwacht hoge of afwijkende waarde ziet?

17. Wat zou u willen weten over uw bloeddruk dat u nu niet uit een losse meting kunt halen?

**Terugkeer naar het dagelijks leven**

18. Zijn er activiteiten waarbij u sinds uw diagnose of behandeling anders bent gaan nadenken over wat veilig is?

19. Hoe ervaart u bewegen, sporten of lichamelijke inspanning?

20. Zijn er andere onderdelen van het dagelijks leven waarop de aortaziekte invloed heeft, bijvoorbeeld werk, gezin, autorijden, reizen of seksualiteit?

21. Welke informatie of ondersteuning mist u om uw gewone leven weer zo goed mogelijk op te pakken?

**Ondersteuning tussen ziekenhuisbezoeken**

22. Zijn er momenten tussen controles waarop u behoefte heeft aan extra informatie of ondersteuning?

23. Wat zou u op zo'n moment willen weten of kunnen doen?

24. Welke informatie over uw gezondheid zou voor u nuttig zijn om over langere tijd te kunnen volgen?

25. Zou het helpen als u bijvoorbeeld klachten, bloeddruk of activiteit over langere tijd zou kunnen terugzien? Waarom wel of niet?

**Digitale ondersteuning**

26. Stel dat er een digitale mogelijkheid zou zijn om u tussen ziekenhuisbezoeken te ondersteunen. Waar zou die u vooral bij moeten helpen?

27. Welke informatie zou zo'n systeem aan u moeten geven?

28. Wat zou zo'n systeem juist niet moeten doen?

29. Wanneer zou een waarschuwing of melding nuttig zijn, en wanneer zou die juist onrust veroorzaken?

30. Als een systeem iets afwijkends signaleert, wat zou u dan willen dat er vervolgens gebeurt?

31. Hoe belangrijk is het voor u om te weten wie uw gegevens kan zien en wat ermee gebeurt?

32. Wat zou het voor u moeilijk maken om zo'n systeem te gebruiken?

33. Hoe zorgen we ervoor dat zo'n systeem ook bruikbaar is voor mensen die minder digitaal vaardig zijn?

**Afsluiting**

34. Als u één ding mocht verbeteren aan de begeleiding van mensen met een aortaziekte tussen ziekenhuiscontroles, wat zou dat zijn?

35. Is er iets belangrijks dat we niet hebben gevraagd?

**Questions (English translation)**

*Translation by a bilingual member of the research team, checked against the original by a second.*

**Opening — living with thoracic aortic disease**

1. If you had to describe in a few words what it is like to live with your aortic condition, which words come to mind?

2. What has changed for you since the diagnosis, dissection or operation?

3. Are there things you now think about, or deal with, differently than before your diagnosis or treatment?

**Uncertainty and symptoms**

4. At which moments in daily life are you most aware that you have a condition of the aorta?

5. What happens when you experience pain, an unusual sensation, or other physical symptoms?

6. How do you decide whether a symptom is something to worry about?

7. When do you decide to contact the hospital or a doctor? What sometimes makes that decision difficult?

8. Are there moments when you feel reassured?

**Check-ups and scans**

9. How do you experience the period between two check-ups or scans?

10. Does that change as a new scan or appointment approaches?

11. What gives you confidence or reassurance after a check-up or scan?

12. Which questions or uncertainties sometimes remain after a check-up?

**Blood pressure**

13. Do you measure your blood pressure at home? How do you experience that?

14. When does measuring your blood pressure help you?

15. Are there situations in which measuring causes unease instead?

16. What do you do when you see an unexpectedly high or abnormal value?

17. What would you like to know about your blood pressure that a single reading cannot tell you?

**Returning to daily life**

18. Are there activities about which you have thought differently, in terms of what is safe, since your diagnosis or treatment?

19. How do you experience movement, exercise or physical exertion?

20. Are there other parts of daily life the condition affects — for example work, family, driving, travel or sexuality?

21. What information or support do you miss in picking up your ordinary life as fully as possible?

**Support between hospital visits**

22. Are there moments between check-ups when you need additional information or support?

23. What would you want to know, or be able to do, at such a moment?

24. Which information about your health would be useful to follow over a longer period?

25. Would it help if you could look back at symptoms, blood pressure or activity over time? Why, or why not?

**Digital support**

26. Suppose a digital option existed to support you between hospital visits. What should it mainly help you with?

27. What information should such a system give you?

28. What should such a system specifically not do?

29. When would a warning or notification be useful, and when would it cause unease?

30. If a system detected something abnormal, what would you want to happen next?

31. How important is it to you to know who can see your data and what happens to it?

32. What would make it difficult for you to use such a system?

33. How do we make sure such a system is also usable for people who are less digitally skilled?

**Closing**

34. If you could improve one thing about the support given to people with an aortic condition between hospital check-ups, what would it be?

35. Is there anything important we have not asked about?
