# Supplementary material for: Co-creating a patient-driven digital monitoring agenda for thoracic aortic disease: a multiphase qualitative study
Source: BMJ Open. 2026 Sep 25;16(9):e121086. doi: 10.1136/bmjopen-2026-121086 (PMC13629912; doi:10.1136/bmjopen-2026-121086)
Supplement: Supplementary file 4 [file bmjopen-16-9-s003.docx]

**Online supplemental file 3**

**Exercise output by session**

*Co-creating a patient-driven digital monitoring agenda for thoracic aortic disease: a multi-phase qualitative study*

**What this file contains**

Each table below reproduces, in full, the items a group produced in response to one stated prompt. Items were written by participants on cards or post-it notes, or collected from the group onto a flip chart, and were pooled during the session without recording which participant contributed which item. No item can therefore be attributed to an individual, and none is presented as a quotation: these are the outputs of structured elicitation exercises, reported under the prompt that generated them.

Complete lists are given rather than selections, so that the reader sees the full distribution of what each exercise produced and can judge whether the prompt could have generated the findings reported in the main text.

Not included here: statements written on flip charts as continuous sentences during discussion, which are the facilitator's contemporaneous compression of speech rather than participants' own text; and the individual before-and-now worksheets, which are individual-level rather than pooled. Both are described in the Methods and neither is reproduced.

Translation from Dutch was carried out by a bilingual member of the research team and checked against the original by a second. Identifying details were removed before analysis.

**A1 — Acute aortic dissection, post-surgery (n=8)**

*Prompt: Write two or three words describing what living with your aorta since the operation means to you	·	Cards*

| **Original (Dutch)** | **English translation** |
| --- | --- |
| onzekerheid · tweede kans · bang dat het weer gebeurt · dankbaar · kwetsbaar · luisteren naar mijn lichaam · controle kwijt · leven · alert · vertrouwen terugkrijgen · elke pijn voel ik · geluk gehad · mijn gezin · scan spannend · voorzichtig · niet meer dezelfde · sterker · ambulance · toekomst? · normaal doen · moe · hartslag voelen · veilig · angst · doorgaan · genieten · controle · wat mag ik nog? · ziekenhuis · weer durven | uncertainty · second chance · afraid it will happen again · grateful · vulnerable · listening to my body · loss of control · life · alert · regaining trust · I feel every pain · I was lucky · my family · scan is nerve-racking · careful · not the same person · stronger · ambulance · future? · acting normal · tired · feeling my heartbeat · safe · fear · carrying on · enjoying things · control · what am I still allowed to do? · hospital · daring again |

**A3 — Elective aortic surgery (n=8)**

*Prompt: What did you run into at home that you had not thought about before the operation? ·	Cards*

| **Original (Dutch)** | **English translation** |
| --- | --- |
| conditie terugkrijgen · bang om zwaar te tillen · vermoeidheid · wanneer weer fietsen? · bloeddruk · werk · slapen · autorijden · hartslag bij traplopen · onzeker wat ik mag · litteken · familie is banger dan ik · sporten weer opbouwen · medicijnen · concentratie · niet patiënt willen blijven · controle-afspraak duurt lang · wie kan ik tussendoor iets vragen? | getting my fitness back · afraid to lift anything heavy · fatigue · when can I cycle again? · blood pressure · work · sleeping · driving · heartbeat when climbing stairs · unsure what I am allowed to do · scar · my family is more frightened than I am · building exercise back up · medication · concentration · not wanting to stay a patient · the follow-up appointment is a long way off · who can I ask something in between? |

**A4 — Aneurysm / bicuspid aortic valve under surveillance (n=7)**

*Prompt: What happens for you between two scans? — after a good result	·	Flip-chart timeline, collected items*

| **Original (Dutch)** | **English translation** |
| --- | --- |
| opluchting · weer door · “tot volgend jaar” · vertrouwen · even vergeten · fijn dat het stabiel is | relief · carrying on · “see you next year” · confidence · forgetting it for a while · glad it is stable |

**A4 — Aneurysm / bicuspid aortic valve under surveillance (n=7)**

*Prompt: What happens for you between two scans? — the months in between	·	Flip-chart timeline, collected items*

| **Original (Dutch)** | **English translation** |
| --- | --- |
| meestal weinig mee bezig · bloeddruk meten · medicijnen · gezond proberen te leven · sporten maar soms twijfel · wat gebeurt er ondertussen? · je voelt niet of hij groeit · liever niet te vaak controleren | mostly not preoccupied with it · measuring blood pressure · medication · trying to live healthily · exercising but sometimes doubting · what is happening in the meantime? · you cannot feel whether it is growing · would rather not check too often |

**A4 — Aneurysm / bicuspid aortic valve under surveillance (n=7)**

*Prompt: What happens for you between two scans? — as the scan approaches	·	Flip-chart timeline, collected items*

| **Original (Dutch)** | **English translation** |
| --- | --- |
| spanning · “zou hij gegroeid zijn?” · slechter slapen · diameter weer in mijn hoofd · oude uitslag terugzoeken · Googlen · bloeddruk vaker meten | tension · “will it have grown?” · sleeping worse · the diameter back in my head · looking up the previous result · Googling · measuring blood pressure more often |

**A4 — Aneurysm / bicuspid aortic valve under surveillance (n=7)**

*Prompt: What happens for you between two scans? — after the scan	·	Flip-chart timeline, collected items*

| **Original (Dutch)** | **English translation** |
| --- | --- |
| “stabiel” = opluchting · hoeveel mm? · waarom andere meting dan vorige keer? · wanneer wordt het opereren? · volgend jaar weer hetzelfde | “stable” = relief · how many mm? · why a different measurement from last time? · when will surgery happen? · same again next year |

**A5 — Mixed: acute, elective, surveillance (n=8)**

*Prompt: Measuring my blood pressure helps me when…	·	Flip chart, collected items*

| **Original (Dutch)** | **English translation** |
| --- | --- |
| ik zie dat het stabiel is · ik weet wat mijn normale waarde is · ik een patroon kan zien · arts zegt hoe vaak ik moet meten · ik zie dat medicijnen werken · geeft gevoel van controle · thuis rustiger dan ziekenhuis · ik kan waarden meenemen naar afspraak · niet alleen momentopname · ik weet wanneer ik iets moet doen | I can see that it is stable · I know what my normal value is · I can see a pattern · my doctor tells me how often to measure · I can see the medication is working · it gives a feeling of control · calmer at home than in hospital · I can take the readings to my appointment · not just a snapshot · I know when I need to act |

**A5 — Mixed: acute, elective, surveillance (n=8)**

*Prompt: Measuring my blood pressure does not help me when…	·	Flip chart, collected items*

| **Original (Dutch)** | **English translation** |
| --- | --- |
| één meting hoog is · ik niet weet waarom hij hoog is · ik meteen opnieuw ga meten · verschillende artsen andere grenzen noemen · apparaat steeds andere waarde geeft · ik gespannen ben · ik alleen rood/groen zie zonder uitleg · ik niet weet wanneer ik moet bellen · ik te vaak kijk · het mijn hele dag gaat bepalen | one reading is high · I don't know why it is high · I immediately measure again · different doctors give different thresholds · the device keeps giving a different value · I am tense · I only see red/green without explanation · I don't know when to call · I look too often · it takes over my whole day |

**A6 — Mixed: acute, elective, surveillance (n=7)**

*Prompt: Suppose something were available between hospital check-ups — what would you find useful? · Post-it notes, clustered by participants*

| **Original (Dutch)** | **English translation** |
| --- | --- |
| uitleg bij klachten · wanneer bellen? · bloeddruk over langere tijd · mijn eigen normale waarden · bewegen · wat mag ik tillen · voorbereiding op controle · vragen opschrijven · medicijnen · uitslagen begrijpelijk · contact als er echt iets verandert · informatie die bij mij past · zwangerschap · werk hervatten · sportadvies · trends zien · geruststelling · niet alles zelf hoeven onthouden · betrouwbare informatie | explanation of symptoms · when to call? · blood pressure over a longer period · my own normal values · exercise · what am I allowed to lift · preparation for the check-up · writing down questions · medication · results in plain language · contact if something really changes · information that fits me · pregnancy · returning to work · exercise advice · seeing trends · reassurance · not having to remember everything myself · reliable information |

**A6 — Mixed: acute, elective, surveillance (n=7)**

*Prompt: …and what absolutely not? ·	Post-it notes, clustered by participants*

| **Original (Dutch)** | **English translation** |
| --- | --- |
| iedere dag vragen hoe het gaat · alarm bij ieder afwijkend getal · alles rood maken · dokter vervangen · nóg een app · ingewikkeld · continu mijn hartslag bekijken · gevoel dat iemand mij 24/7 controleert · gegevens delen zonder te weten met wie · standaardadvies voor iedereen · steeds herinnerd worden dat ik patiënt ben | being asked every day how I am · an alarm for every abnormal value · making everything red · replacing the doctor · yet another app · complicated · constantly watching my heart rate · the feeling that someone is checking on me 24/7 · sharing data without knowing who with · standard advice for everyone · being constantly reminded that I am a patient |

**B1 — Women across life stages (n=7)**

*Prompt: At which moments in your life do you notice that your aortic condition affects the choices you make? ·	Cards*

| **Original (Dutch)** | **English translation** |
| --- | --- |
| zwangerschap · kinderwens · sporten · werk · kinderen optillen · vermoeidheid · anticonceptie · bang om zwanger te worden · mantelzorg · overgang · bloeddruk · gezin belasten · carrière · erfelijkheid · medicijnen en zwangerschap · wat kan mijn lichaam aan? · seksualiteit · toekomst plannen · schuldgevoel naar kinderen · verschillende adviezen | pregnancy · wish to have children · exercise · work · lifting children · fatigue · contraception · afraid of becoming pregnant · caring for a relative · menopause · blood pressure · being a burden on the family · career · heritability · medication and pregnancy · what can my body take? · sexuality · planning for the future · guilt towards my children · conflicting advice |

**B1 — Women across life stages (n=7)**

*Prompt: What do you currently get answers to? ·	Flip chart, collected items*

| **Original (Dutch)** | **English translation** |
| --- | --- |
| diameter aorta · uitslag scan · bloeddruk · medicijnen · wanneer volgende controle · operatie-indicatie · algemene sportadviezen | aortic diameter · scan result · blood pressure · medication · when the next check-up is · indication for surgery · general exercise advice |

**B1 — Women across life stages (n=7)**

*Prompt: And what remains unclear? ·	Flip chart, collected items*

| **Original (Dutch)** | **English translation** |
| --- | --- |
| Kan ik veilig zwanger worden? · Wie bespreekt zwangerschap met mij? · Wanneer moet ik dit zelf ter sprake brengen? · Mag ik mijn kind tillen? · Wat betekent mijn aandoening voor de overgang? · Hoe combineer ik werk en herstel? · Hoe zwaar mag ik trainen? · Welke medicijnen mogen bij kinderwens? · Wat vertel ik mijn kinderen? · Is dit erfelijk? · Geldt algemeen advies ook voor mij? | Can I safely become pregnant? · Who discusses pregnancy with me? · When should I raise this myself? · May I lift my child? · What does my condition mean at menopause? · How do I combine work and recovery? · How hard may I train? · Which medications are compatible with a wish to conceive? · What do I tell my children? · Is this heritable? · Does general advice apply to me too? |

**B2 — Accessibility and digital literacy (n=6)**

*Prompt: What makes digital care easy for you? ·	Cards*

| **Original (Dutch)** | **English translation** |
| --- | --- |
| telefoon heb ik altijd bij me · grafiek is duidelijk · herinnering medicijnen · grote letters · één plek voor informatie · Nederlands én andere taal · simpele uitleg · zelf kunnen kiezen · familie mee laten kijken · bloeddruk automatisch opslaan | I always have my phone with me · a clear graph · medication reminders · large type · one place for information · Dutch and another language · simple explanation · being able to choose myself · letting family look with me · blood pressure saved automatically |

**B2 — Accessibility and digital literacy (n=6)**

*Prompt: And what makes it difficult? ·	Cards*

| **Original (Dutch)** | **English translation** |
| --- | --- |
| te veel apps · wachtwoorden · DigiD · medische woorden · kleine letters · grafieken snap ik niet altijd · updates · meldingen · bang iets verkeerd in te vullen · geen nieuwe telefoon · alles alleen Nederlands · wie ziet mijn gegevens? · geen persoonlijk contact · te veel tekst | too many apps · passwords · national digital identity login · medical vocabulary · small type · I don't always understand graphs · updates · notifications · afraid of entering something wrong · no new phone · everything in Dutch only · who sees my data? · no personal contact · too much text |

**B2 — Accessibility and digital literacy (n=6)**

*Prompt: Make it usable for everyone	·	Flip chart, collected items*

| **Original (Dutch)** | **English translation** |
| --- | --- |
| niet verplicht · eenvoudige taal · uitleg met beeld · voorlezen mogelijk · verschillende talen · familie/mantelzorger kunnen betrekken · ook zonder smartwatch · niet iedere dag hoeven invullen · telefoon moet voldoende zijn · menselijke hulp moet blijven · patiënt bepaalt meldingen · duidelijk waar gegevens naartoe gaan | not compulsory · plain language · explanation with images · read-aloud option · different languages · able to involve family or a carer · usable without a smartwatch · not having to fill something in every day · a phone should be enough · human help must remain · the patient decides on notifications · clear where the data goes |

**C — Co-design workshop: 6 patients, 3 healthcare professionals (n=9)**

*Prompt: What could help between hospital visits with: “I don't know whether a symptom matters”	·	Post-it notes*

| **Original (Dutch)** | **English translation** |
| --- | --- |
| klachten kunnen beschrijven · vragen stap voor stap · hoe lang bestaat het? · wordt het erger? · eerdere klachten vergelijken · wanneer bellen · geen diagnose door app · samenvatting voor arts | being able to describe symptoms · questions step by step · how long has it been there? · is it getting worse? · comparing with earlier symptoms · when to call · no diagnosis by app · a summary for the doctor |

**C — Co-design workshop (n=9)**

*Prompt: What could help with: “One high blood pressure reading makes me anxious”	·	Post-it notes*

| **Original (Dutch)** | **English translation** |
| --- | --- |
| niet reageren op één waarde · week/maand laten zien · trend · gemiddelde · context: stress / sport / ziekte · meetinstructie · persoonlijk meetplan · wanneer opnieuw meten · wanneer contact opnemen | not reacting to a single value · showing a week or a month · trend · average · context: stress / exercise / illness · measurement instructions · a personal measurement plan · when to measure again · when to make contact |

**C — Co-design workshop (n=9)**

*Prompt: What could help with: “I don't know what safe activity is”	·	Post-it notes*

| **Original (Dutch)** | **English translation** |
| --- | --- |
| activiteit + klachten combineren · persoonlijke doelen · herstel na operatie · advies per fase · niet alleen stappen tellen · sport bespreken bij controle · vertrouwen opbouwen · terugkeer naar werk meenemen | combining activity with symptoms · personal goals · recovery after surgery · advice by stage · not just counting steps · discussing exercise at the check-up · building confidence · including return to work |

**C — Co-design workshop (n=9)**

*Prompt: What could help with: “I get information in different places”	·	Post-it notes*

| **Original (Dutch)** | **English translation** |
| --- | --- |
| informatie bij elkaar · persoonlijk dossier · zwangerschap · werk · sport · medicijnen · erfelijkheid · alleen tonen wat voor mij relevant is | information in one place · a personal record · pregnancy · work · exercise · medication · heritability · showing only what is relevant to me |

**C — Co-design workshop (n=9)**

*Prompt: You have five stickers each. What must a first version absolutely provide? (ranked by vote, highest first)	·	Flip chart after voting*

| **Original (Dutch)** | **English translation** |
| --- | --- |
| Bloeddruk als trend weergeven · Hulp bij beschrijven/contextualiseren klachten · Duidelijk wanneer contact opnemen · Persoonlijke informatie/educatie · Activiteit in context · PROMs / hoe gaat het over tijd? · Medicatieherinnering · Continue hartslagmonitoring · Automatische diagnose | Displaying blood pressure as a trend · Help in describing and contextualising symptoms · Clarity on when to make contact · Personalised information and education · Activity in context · Patient-reported outcomes over time · Medication reminders · Continuous heart-rate monitoring · Automated diagnosis |

**C — Co-design workshop (n=9)**

*Prompt: What should happen when something deviates? (jointly drawn pathway)	·	Flip chart*

| **Original (Dutch)** | **English translation** |
| --- | --- |
| meting / klacht → eerst context → patroon of eenmalig? → uitleg patiënt → zo nodig opnieuw meten → duidelijke actie; zelf volgen → contact poli → urgente beoordeling. Voorwaarden: geen zwart doosje · patiënt moet weten waarom melding komt · niet iedere afwijking naar ziekenhuis · spoed blijft spoed · verantwoordelijkheid duidelijk · wie ontvangt melding? · geen verwachting van 24/7 monitoring | measurement / symptom → context first → pattern or one-off? → explanation to the patient → re-measure if needed → a defined action; self-monitoring → contact with the outpatient clinic → urgent assessment. Conditions: no black box · the patient must know why an alert was generated · not every deviation goes to the hospital · emergencies remain emergencies · responsibility made clear · who receives the alert? · no expectation of 24/7 monitoring |

**D — Member-check validation (n=9)**

*Prompt: For each domain: do you recognise it, do you doubt it, is something missing? — Managing uncertainty and fear of recurrence	·	Cards*

| **Original (Dutch)** | **English translation** |
| --- | --- |
| herken ik · vooral na dissectie · angst verandert met tijd · niet iedereen wil méér monitoring · monitoring kan ook angst geven | I recognise this · especially after dissection · fear changes with time · not everyone wants more monitoring · monitoring can also generate fear |

**D — Member-check validation (n=9)**

*Prompt: — Symptom interpretation	·	Cards*

| **Original (Dutch)** | **English translation** |
| --- | --- |
| heel herkenbaar · belangrijk · “wanneer moet ik bellen?” ontbreekt vaak · app mag nooit zeggen dat pijn veilig is · voorbereiding op contact is goed | very recognisable · important · “when should I call?” is often missing · an app must never say that pain is safe · preparing for contact is good |

**D — Member-check validation (n=9)**

*Prompt: — Longitudinal blood pressure patterns	·	Cards*

| **Original (Dutch)** | **English translation** |
| --- | --- |
| ja · liever trend · losse waarde geeft stress · persoonlijk doel belangrijk · niet te vaak meten | yes · prefer a trend · a single value causes stress · a personal target matters · do not measure too often |

**D — Member-check validation (n=9)**

*Prompt: — Life participation	·	Cards*

| **Original (Dutch)** | **English translation** |
| --- | --- |
| heel belangrijk · sport · werk · zwangerschap · gezin · herstel is meer dan scan · graag persoonlijk advies | very important · exercise · work · pregnancy · family · recovery is more than the scan · personal advice would be welcome |

**D — Member-check validation (n=9)**

*Prompt: — Accessibility and proportionality	·	Cards*

| **Original (Dutch)** | **English translation** |
| --- | --- |
| absoluut behouden · keuze patiënt · niet verplicht · menselijke zorg behouden · eenvoudig · privacy · niet iedereen hetzelfde laten meten | definitely keep this · the patient's choice · not compulsory · preserve human care · simple · privacy · do not ask everyone to measure the same things |

**D — Member-check validation (n=9)**

*Prompt: If this system existed tomorrow: when would it help you? · Flip chart, collected items*

| **Original (Dutch)** | **English translation** |
| --- | --- |
| als ik twijfel · tussen controles · voor afspraak · bij verandering bloeddruk · terugkeer naar sport · overzicht over tijd · betrouwbare informatie · vragen voorbereiden · als duidelijk is wat ik moet doen | when I am unsure · between check-ups · before an appointment · when blood pressure changes · returning to exercise · an overview over time · reliable information · preparing questions · when it is clear what I should do |

**D — Member-check validation (n=9)**

*Prompt: And when would it work against you? ·	Flip chart, collected items*

| **Original (Dutch)** | **English translation** |
| --- | --- |
| als ik continu meldingen krijg · als iedere afwijking rood wordt · als ik dagelijks moet invullen · als arts denkt dat app contact vervangt · als ik niet weet wie meekijkt · als het angst groter maakt · als advies niet persoonlijk is · als ik het niet kan uitzetten | if I get constant notifications · if every deviation turns red · if I have to fill something in daily · if the doctor thinks the app replaces contact · if I don't know who is looking · if it increases anxiety · if the advice is not personal · if I cannot switch it off |
